# Health Risk Behaviours by Immigrants’ Duration of Residence: A Systematic Review and Meta-Analysis

**DOI:** 10.3389/ijph.2022.1604437

**Published:** 2022-08-05

**Authors:** Sol P. Juárez, Helena Honkaniemi, Nina-Katri Gustafsson, Mikael Rostila, Lisa Berg

**Affiliations:** ^1^ Centre for Health Equity Studies (CHESS), Stockholm University/Karolinska Institutet, Stockholm, Sweden; ^2^ Department of Public Health Sciences, Faculty of Social Sciences, Stockholm University, Stockholm, Sweden

**Keywords:** alcohol, diet, physical activity (PA), smoking, substance use, health risk behaviours, length of stay, immigrants

## Abstract

**Objectives:** The aim was to systematically review and synthesise international evidence on changes in health risk behaviours by immigrants’ duration of residence.

**Methods:** We searched literature databases for peer-reviewed quantitative studies published from 2000 to 2019, examining alcohol, drug and tobacco use; physical inactivity; and dietary habits by duration of residence.

**Results:** Narrative synthesis indicated that immigrants tend to adopt health risk behaviours with longer residence in North America, with larger variation in effect sizes and directionality in other contexts. Random-effects meta-analyses examining the pooled effect across all receiving countries and immigrant groups showed lower odds of smoking (OR 0.54, 0.46–0.63, *I* ^2^ = 68.7%) and alcohol use (OR 0.61, 0.47–0.75, *I* ^2^ = 93.5%) and higher odds of physical inactivity (OR 1.71, 1.40–2.02, *I* ^2^ = 99.1%) among immigrants than natives, but did not provide support for a universal trend by duration of residence.

**Conclusion:** Findings suggest that duration of residence could serve as an effective instrument to monitor immigrants’ health changes. However, differences in receiving country contexts and immigrant populations’ composition seem to be important to predict the level and direction of behavioural change.

**Systematic Review Registration:** https://www.crd.york.ac.uk/prospero/, PROSPERO CRD42018108881.

## Introduction

Health risk behaviours, including alcohol, drug and tobacco use, physical inactivity and poor dietary habits are responsible for a myriad of adverse outcomes [1, 2]. Due to the uneven distribution of these behaviours across social groups, they also contribute to the maintenance of health inequalities [3–5]. The adoption of health risk behaviours has commonly been hypothesised to explain why immigrants frequently experience health deterioration with increasing time in their receiving country, often to converge with native risks [6].

Although the literature on immigrants’ health risk behaviours by duration of residence is extensive, there is a general lack of synthesised evidence. To date, the only systematic reviews that have examined changes in immigrants’ health risk behaviours in the receiving context—albeit for specific health risk behaviours, immigrant populations and receiving country contexts [7–16]—have exclusively focused on the role of acculturation (the process by which immigrants assimilate and gradually adopt norms, values and characteristics of the majority population in the receiving country). Besides applying an unequivocal theoretical approximation to interpreting findings (a cultural lens), this approach has favoured the use of specific instruments (acculturation measures) and reduced the use of others within the same domain (including for duration of residence). Furthermore, this practice has limited the synthesis of evidence beyond the acculturation framework, which in turn limits the capacity to propose and discuss alternative models, such as the role of social inequalities in health behaviours. Thus, despite the availability of an extensive literature on changes in immigrants’ health risk behaviours by duration of residence, to our knowledge, there have been no previous systematic evaluations.

This systematic review aimed to comprehensively synthesise international evidence on changes in health risk behaviours, including alcohol, drug and tobacco use, physical activity and diet, by immigrants’ duration of residence, with immigrant and native reference populations. Through meta-analysis, we additionally aimed to investigate whether universal patterns of health behavioural changes exist across heterogeneous immigrant populations and country contexts.

## Methods

### Search Strategy and Selection Criteria

This systematic review and meta-analysis was conducted in compliance with the Preferred Reporting Items for Systematic Reviews and Meta-Analyses (PRISMA) guidelines [17]. PubMed/MEDLINE, Web of Science, and ProQuest databases were systematically searched for peer-reviewed English-language articles published online between January 1st, 2000 and December 31st, 2019. Search strings were developed over multiple iterations (Supplementary Table S1).

Studies were included if they examined international immigrants aged 18–64 years at the time of assessment (including individuals who migrated as children), by duration of residence (with a reference group in the receiving country), with a health risk behaviour outcome, including alcohol, drug (i.e., illicit substances) and tobacco (i.e., cigarettes, smokeless tobacco products) use; physical inactivity; and poor dietary habits. We did not distinguish by countries or regions of origin and destination; reasons for migration; or native or immigrant reference groups. Studies on internal migrants; temporary migrants; circular and return migrants; and minority groups indistinguishable by their foreign-birth status were excluded, as were studies focusing on transitional life periods, i.e., adolescence or retirement. Only studies utilising quantitative or mixed methods with observational data were considered. Qualitative studies and grey literature were excluded.

Using the Covidence systematic review online management tool, two authors independently screened and selected papers by title and abstract. Full-length articles were then reviewed by four authors in review pairs.

The study protocol was registered in PROSPERO (no. CRD42018108881), then peer-reviewed and published with open access, detailing the search strategy and selection process [18].

### Data Analysis

Relevant data from the selected studies was extracted in review pairs using a piloted and standardised data extraction form. Extracted data included sample characteristics (e.g., sample size, age range, sex proportion, socioeconomic information) and details about exposure and outcome measures, analytical approaches, effect measures and controls. Each study was independently and systematically assessed for quality by two authors, using modified versions of the Newcastle-Ottawa Scale for cross-sectional studies (based on original case-control version) and cohort studies [19], and checked by a third author for completeness and accuracy.

We conducted a narrative synthesis to explore and summarise patterns of health risk behaviours by duration of residence. In the narrative synthesis, particular attention was given to studies with native reference groups to explore patterns of convergence and divergence with behaviours of native-born populations. We examined findings for different health risk behaviours separately and, given sufficient data from the included studies, by sex, immigrant region of origin and study (i.e., receiving) region. The narrative synthesis followed the Synthesis Without Meta-analysis [20] and PRISMA guidelines.

Thereafter, random-effects meta-analyses [21] were performed using the metan command in Stata version 13 [22]. Results were presented in Forest plots to visualise changes in health risk behaviours by immigrants’ duration of residence, relative to native-born populations. Summary estimates were used to broadly compare health risk behaviours between immigrants and natives, and I^2^ statistics to indicate the proportion of variance attributable to study heterogeneity (i.e., due to differences in duration of residence measures) [23]. By treating the native-born populations as a homogeneous reference group, these analyses permitted us to evaluate whether there was a universal pattern of health behavioural changes by duration of residence comparable to other empirical generalisations, such as the healthy immigrant paradox [24]. Summary estimates are presented as Odds Ratios (ORs) with 95% Confidence Intervals (CI) and plotted as log ORs to facilitate visualisation. Unadjusted Risk Ratios were transformed into ORs when enough information was provided in the studies. Separate analyses were conducted for unadjusted and adjusted estimates. When several model specifications were available, we extracted information from minimally-adjusted models, i.e., adjusting for sex, age and socioeconomic information, to avoid over-adjustment.

Subgroup meta-analyses were conducted by country or region of birth, receiving country and sex, when data allowed. Subgroup analyses by type of alcohol use (i.e., regular vs. binge drinking) and physical inactivity (i.e., leisure- vs. non-leisure-time) were not possible due to insufficient data for statistical pooling. Finally, we conducted subgroup analyses by different categorisations of duration of residence to assess the sensitivity of the results. In reporting meta-analysis results, Meta-analysis of Observational Studies in Epidemiology (MOOSE) Group recommendations were followed [25].

Any conflicts in the review, extraction or quality rating processes were resolved through discussion with other review team members. Study authors were contacted if clarification was needed.

## Results

**FIGURE 1:**
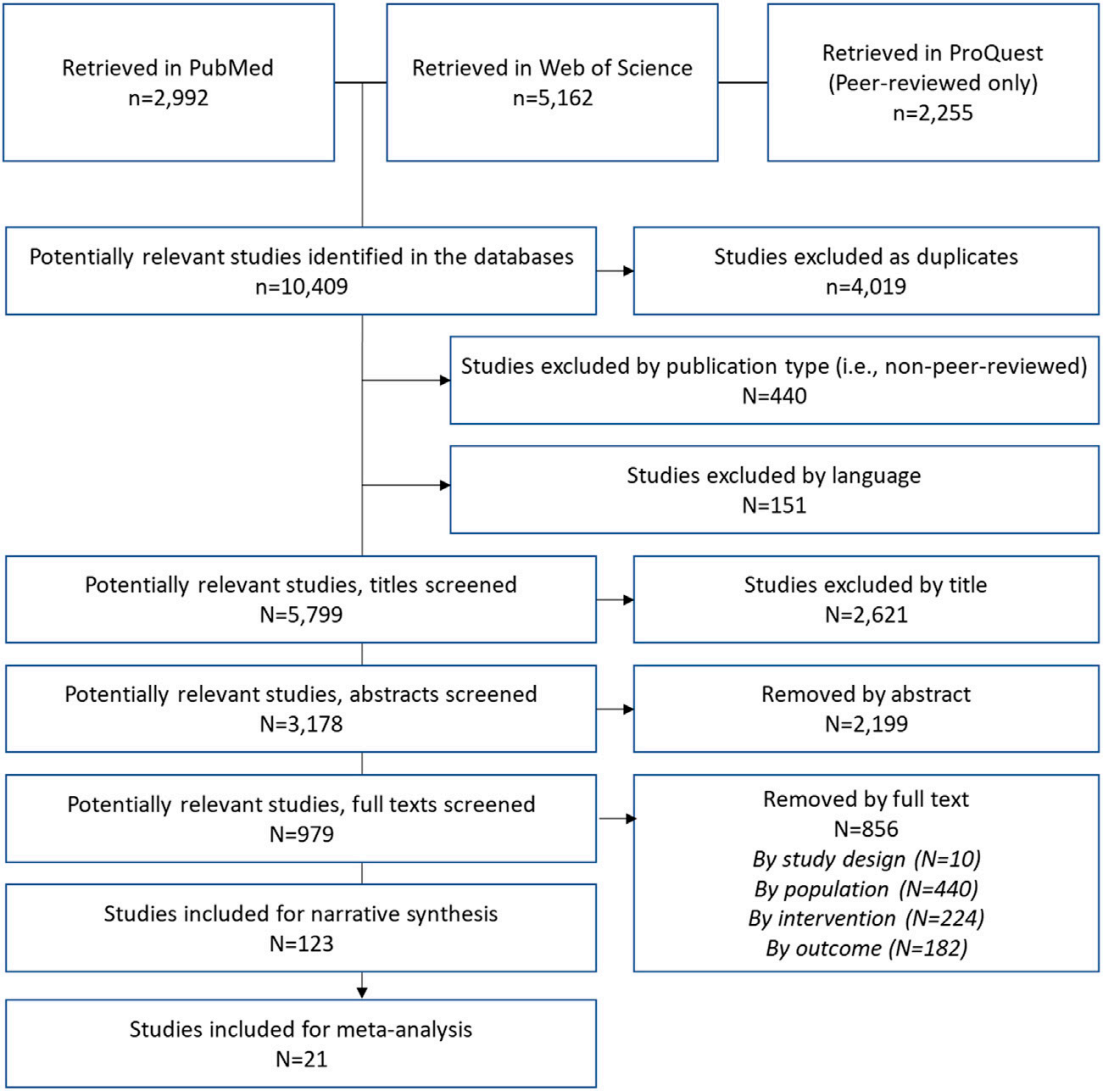
Selection process (Sweden, 2022).

The database searches yielded 10,409 records (Figure 1), of which 4,019 were removed as duplicates, 440 by publication type and 151 by language. Non-relevant studies were excluded by title (*n* = 2,621), abstract (*n* = 2,199) and full text (*n* = 856), with a final count of 123 included studies (Supplementary Table S2) [26–148].

Studies examined tobacco (*n* = 58), alcohol (*n* = 31) and drug use (*n* = 10); substance use diagnoses or dependence (*n* = 11); physical inactivity (*n* = 49); and diet (*n* = 17). Most studies were cross-sectional (*n* = 117), with a few longitudinal studies (*n* = 6), based on large-scale or study-specific surveys and interviews from 1988 to 2015. More than half of the studies (56%) were rated 75% or better (Supplementary Tables S3, S4). Included studies were from North America, including the USA (*n* = 81) and Canada (*n* = 12); Europe, including Finland (*n* = 1), France (*n* = 2), Germany (*n* = 7), Ireland (*n* = 1), the Netherlands (*n* = 6), Norway (*n* = 1), Spain (*n* = 4), Sweden (*n* = 1) and the UK (*n* = 7); as well as Australia (*n* = 6) and Israel (*n* = 1). Immigrant origins were commonly unspecified. Specified regions of origin can be broadly summarised as Asian (*n* = 41), African/Afro-Caribbean (*n* = 19), Latino/Hispanic (*n* = 31), European (*n* = 5) and Middle Eastern (*n* = 8). Some studies presented sex-stratified (*n* = 35) or sex-specific findings for men (*n* = 13) and women (*n* = 12). See Supplementary Table S5 for index of study characteristics.

Findings from the narrative synthesis of the 123 included studies are described below, with detailed tables provided as Supplementary Tables S6–S20.

### Tobacco Use

With one exception [117], all included tobacco-related studies referred to smoking. In North America, most studies revealed increased risks of smoking with longer residence among general immigrant populations [67, 89, 96, 102, 109, 128, 146], as well as Latino/Hispanic [71, 119] and African/Afro-Caribbean [44, 127] immigrants specifically. Findings among Asian immigrants ranged from increasing [100, 103, 117] to decreasing [34, 72, 78, 115] risks, or no trend at all [50, 90, 133]. Asian immigrant men in particular had decreased risks of smoking with longer residence [31, 65, 70, 81, 106, 123], while findings for Latino/Hispanic men were less conclusive [39, 106, 123, 124]. Increased risks of smoking with longer residence appeared for immigrant women of unspecified origin [32, 52, 89, 100, 102, 144], and of African [74], Asian [31, 53, 106, 117, 123, 145] and Latino/Hispanic origin [106, 123].

For immigrants residing in Europe, increased risks of smoking were apparent with longer residence, regardless of country of origin or destination [40, 45, 82, 131]. Findings were mixed among men, with African men showing increased risks [85, 125], Eastern European men no change or decreased risks [125] and Middle Eastern men varied risks of smoking by longer residence [120–122, 125]. Immigrant women largely had increased risks of smoking with longer residence [75, 85, 121, 122, 125]. Studies from Australia were generally inconclusive with regards to directionality of risks [43, 76, 80, 137].

Among North American studies with native-born references, risks generally converged to native levels [32, 44, 102, 119, 123, 128], with some evidence of divergence [34, 39, 60, 123] or unclear patterns relative to natives [39, 93, 109, 114]. European evidence on convergence was more inconclusive, varying by country of origin and destination [64, 85, 131].

**FIGURE 2:**
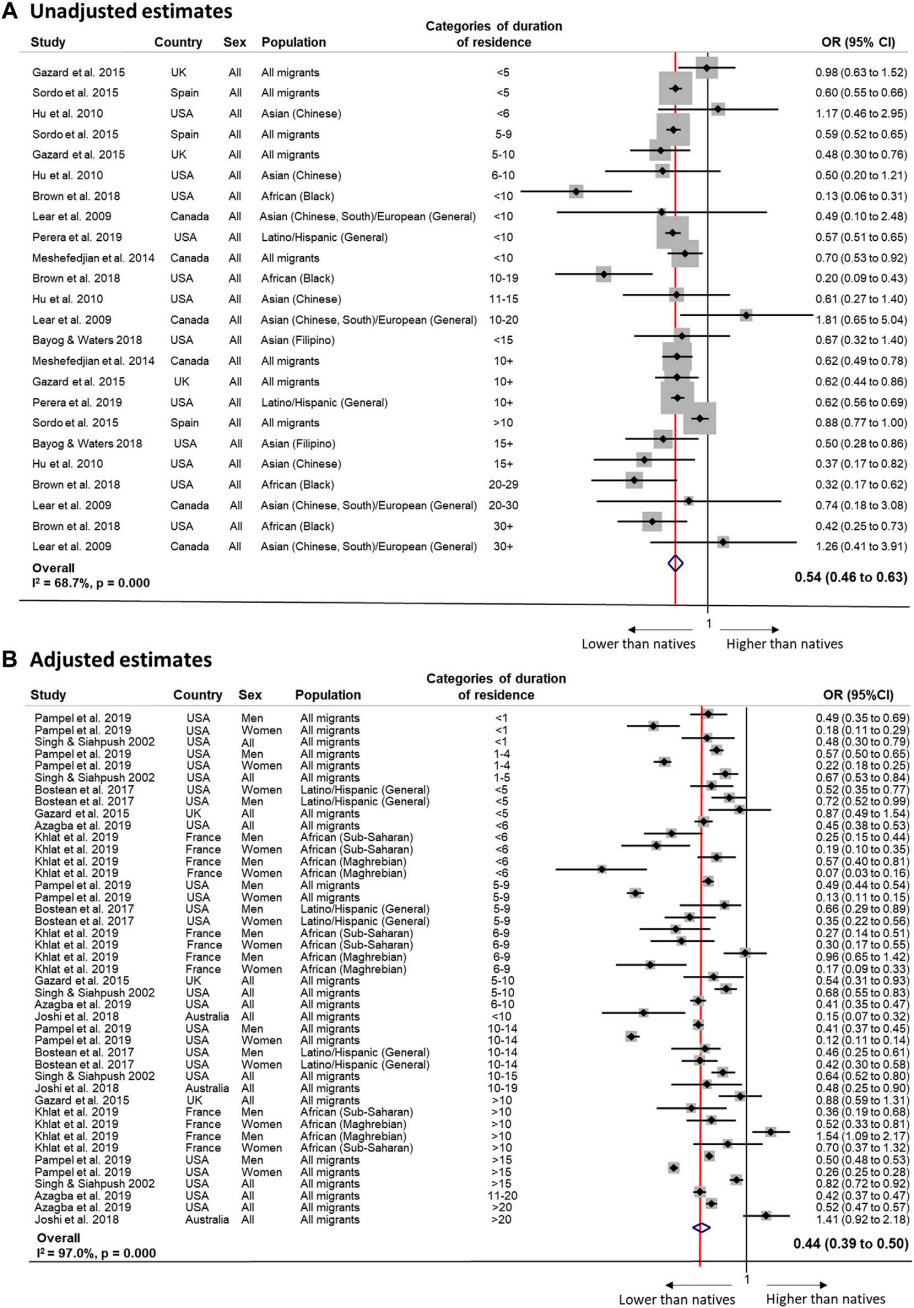
Random-effects meta-analysis of unadjusted (Panel **(A)**) and adjusted (Panel **(B)**) estimates of the association between tobacco use and duration of residence among migrants compared to the native population (Sweden, 2022). Panel **(A)**: <5 years 0.73 (0.38 to 1.07), I^2^ = 62.6%, *p* 0.102 (2 estimates); ≥5 years 0.52 (0.43 to 0.62); I^2^ = 81.7%, *p* 0.000 (21 estimates); <10 years 0.54 (0.43 to 0.66); I^2^ = 83.4%, *p* 0.000 (10 estimates); ≥10 years 0.54 (0.42 to 0.67); I^2^ = 77.6%, *p* 0.000 (14 estimates); <15 years 0.57 (0.48 to 0.66); I^2^ = 83.8%, *p* 0.000 (18 estimates); ≥15 years 0.40 (0.27 to 0.53); I^2^ = 0%, *p* 0.829 (6 estimates). By host country: USA <5 years N/a; ≥5 years 0.42 (0.29 to 0.56); I^2^ = 85.5%, *p* 0.000 (11 estimates); <10 years 0.43 (0.01 to 0.85), I^2^ = 94.8%, *p* 0.000 (3 estimates); ≥10 years 0.44 (0.28 to 0.60); I^2^ = 76.0%, *p* 0.000 (8 estimates); <15 years 0.45 (0.27 to 0.63); I^2^ = 89.3%, *p* 0.000 (8 estimates); ≥15 years 0.40 (0.27 to 0.53); I^2^ = 0.0%, *p* 0.802 (4 estimates). Panel **(B)**: <5 years 0.49 (0.34 to 0.65), I^2^ = 93.7%, *p* 0.000 (9 estimates); ≥5 years 0.46 (0.39 to 0.54); I^2^ = 98.2%, *p* 0.000 (26 estimates); <10 years 0.38 (0.30 to 0.47); I^2^ = 95.1%, *p* 0.000 (23 estimates); ≥10 years 0.52 (0.42 to 0.62); I^2^ = 98.5%, *p* 0.000 (17 estimates); <15 years 0.42 (0.36 to 0.49); I^2^ = 95.9%, *p* 0.000 (37 estimates); ≥15 years 0.54 (0.39 to 0.69); I^2^ = 98.8%, *p* 0.000 (6 estimates). By gender: Men <5 years 0.57 (0.49 to 0.66), I^2^ = 17.2%, *p* 0.299 (3 estimates); ≥5 years 0.48 (0.41 to 0.55); I^2^ = 81.1%, *p* 0.000 (9 estimates); <10 years 0.51 (0.41 to 0.60); I^2^ = 74.4%, *p* 0.000 (9 estimates); ≥10 years 0.48 (0.38 to 0.58); I^2^ = 86.6%, *p* 0.000 (5 estimates); <15 years N/a (1 estimates); ≥15 years 0.50 (0.42 to 0.58); I^2^ = 79.3%, *p* 0.000 (13 estimates). Women <5 years 0.26 (0.15 to 0.37), I^2^ = 76.7%, *p* 0.014 (3 estimates); ≥5 years 0.25 (0.18 to 0.33); I^2^ = 96.4%, *p* 0.000 (9 estimates); <10 years 0.20 (0.15 to 0.26); I^2^ = 82.4%, *p* 0.000 (9 estimates); ≥10 years 0.30 (0.19 to 0.42); I^2^ = 97.9%, *p* 0.000 (5 estimates); <15 years 0.21 (0.17 to 0.26); I^2^ = 85.9%, *p* 0.000 (13 estimates; ≥15 years N/a (1 estimates). By origin: “All migrants” <5 years 0.47 (0.27 to 0.63), I^2^ = 94.5%, *p* 0.000 (7 estimates); ≥5 years 0.46 (0.37 to 0.56); I^2^ = 99.0%, *p* 0.000 (14 estimates); <10 years 0.39 (0.28 to 0.51); I^2^ = 97.3%, *p* 0.000 (11 estimates); ≥10 years 0.50 (0.39 to 0.62); I^2^ = 99.0%, *p* 0.000 (11 estimates); <15 years 0.43 (0.34 to 0.51); I^2^ = 97.6%, *p* 0.000 (19 estimates); ≥15 years 0.54 (0.39 to 0.69); I^2^ = 98.8%, *p* 0.000 (6 estimates). By host country: USA <5 years 0.47 (0.31 to 0.63), I^2^ = 94.3%, *p* 0.000 (8 estimates); ≥5 years 0.43 (0.34 to 0.529); I^2^ = 98.9%, *p* 0.000 (15 estimates); <10 years 0·44 (0.33 to 0.55); I^2^ = 96.9%, *p* 0.000 (13 estimates); ≥10 years 0.45 (0.34 to 0.57); I^2^ = 99.1%, *p* 0.000 (10 estimates); <15 years 0.44 (0.36 to 0.52); I^2^ = 97.5%, *p* 0.000 (20 estimates); ≥15 years 0.50 (0.35 to 0.65); I^2^ = 99·9%, *p* 0.000 (5 estimates).

Meta-analyses showed lower odds of smoking among immigrants compared to natives regardless of model specification (unadjusted OR 0.54, 95% CI 0.46–0.63, *I* ^2^ = 68.7%; adjusted OR 0.44, 95% CI 0.39–0.50, *I* ^2^ = 97.0%; Figure 2), but with no clear patterns of change by duration of residence.

### Alcohol Use

Immigrants showed increased risks of mild-to-moderate alcohol use with longer residence in North America [77, 102, 109], but unclear trends in heavy alcohol use [28, 35, 48]. Latino/Hispanic immigrants generally decreased their regular alcohol use with longer residence [38, 46], while Asian immigrants’ direction of risks varied widely [101, 115, 116, 129, 133]. There was evidence of increased risks of regular [102, 147] and heavy drinking with longer residence [100, 102] for immigrant men. Studies on immigrant women indicated increased risks with longer duration for regular [47, 61, 69, 102, 145] and heavy drinking [100, 102], especially among Latina/Hispanic women [47, 61]. There was little to no consensus on the direction of alcohol use patterns by residence in European countries. General, African, Latino/Hispanic, Eastern European and Middle Eastern immigrants showed both increased [26, 64, 125, 131] and decreased alcohol use [30, 45, 125, 131], depending on the receiving country.

In North America, immigrants consistently converged towards native levels of alcohol use [47, 77, 101, 102, 109], with limited evidence of diverging trends [38]. European studies with native reference groups failed to show consistent patterns of convergence/divergence [64, 131].

**FIGURE 3:**
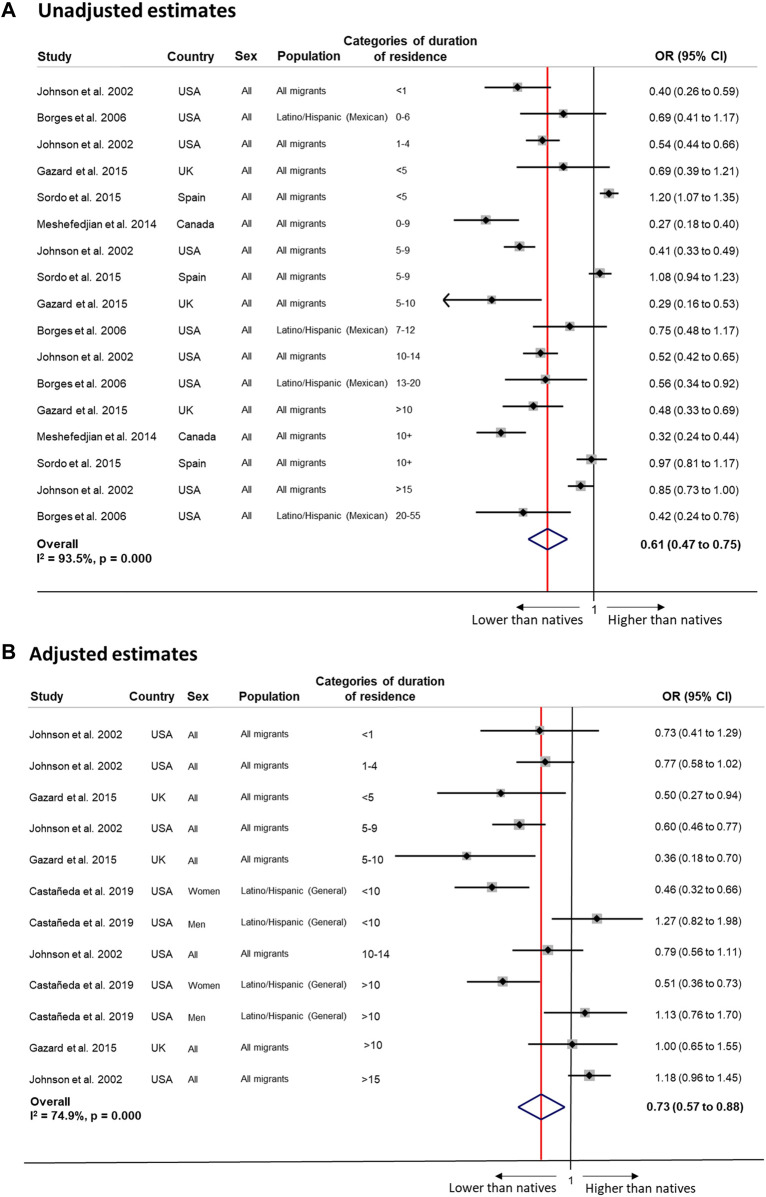
Random-effects meta-analysis of unadjusted (Panel **(A)**) and adjusted (Panel **(B)**) estimates of the association between alcohol and duration of residence among migrants compared to the native population (Sweden, 2022). Panel **(A)**: <5 years 0.71 (0.31 to 1.11), I^2^ = 95.9%, *p* 0.000 (4 estimates); ≥5 years 0.60 (0.44 to 0.77); I^2^ = 92.4%, *p* 0.000 (11 estimates); <10 years 0.65 (0.41 to 0.90); I^2^ = 96.0%, *p* 0.000 (8 estimates); ≥10 years 0.59 (0.40 to 0.78); I^2^ = 90.3%, *p* 0.000 (7 estimates); <15 years 0.60 (0.45 to 0.76); I^2^ = 93.8%, *p* 0.000 (15 estimates); ≥15 years 0.65 (0.23 to 1.07); I^2^ = 87.9%, *p* 0.000 (2 estimates). By host country: USA <5 years 0.48 (0.35 to 0.61), I^2^ = 48.8%, *p* 0.162 (2 estimates); ≥5 years 0.58 (0.41 to 0.75), I^2^ = 85.0%, *p* 0.000 (6 estimates); <10 years 0.46 (0.37 to 0.56); I^2^ = 46.7%, *p* 0.131 (4 estimates); ≥10 years 0.60 (0.39 to 0.81); I^2^ = 81.8%, *p* 0.001 (4 estimates); <15 years 0.49 (0.42 to 0.57); I^2^ = 38.7%, *p* 0.134 (7 estimates); ≥15 years 0.65 (0.23 to 1.07); I^2^ = 87.9%, *p* 0.000 (2 estimates). Panel **(B)**: <5 years 0.70 (0.53 to 0.86), I^2^ = 00.0%, *p* 0.412 (3 estimates); ≥5 years 0.75 (0.56 to 0.95); I^2^ = 80.9%, *p* 0.000 (9 estimates); <10 years 0.64 (0.48 to 0.79); I^2^ = 53.3%, *p* 0.058 (6 estimates); ≥10 years 0.90 (0.60 to 1.20); I^2^ = 81.3%, *p* 0.000 (5 estimates). By origin: “All migrants” <10 years: 0.64 (0.53 to 0.76); I^2^ = 0.0%, *p* 0.489 (4 estimates); ≥10 years 1.00 (0.73 to 1.26); I^2^ = 53.6%, *p* 0.116 (3 estimates). By host country: USA <5 years N/a; ≥5 years 0.79 (0.57 to 1.01); I^2^ = 82.7%, *p* 0.000 (7 estimates); <10 years 0.67 (0.49 to 0.85); I^2^ = 61.1%, *p* 0.036 (5 estimates); ≥10 years 0.88 (0.53 to 1.24); I^2^ = 85.4%, *p* 0.000 (4 estimates).

Pooled meta-analysis estimates showed lower odds of alcohol use among immigrants compared to natives (unadjusted OR 0.61, 95% CI 0.47–0.75, *I* ^2^ = 93.5%; adjusted OR 0.73, 95% CI 0.57–0.88, *I* ^2^ = 74.9%; Figure 3). Changes by duration of residence were observed in analyses of “All immigrants”, using 10 years as a cut-off (<10 years: OR 0.64, 95% CI 0.53 to 0.76, *I* ^*2*^ = 0.0%, *p* = 0.489 (4 estimates); >10 years: OR 1.00, 95% CI 0.73 to 1.26, *I* ^*2*^ = 53.6%, *p* = 0.116 (3 estimates)), suggesting convergence to native levels.

### Drug Use

Increased risks of drug use with longer residence in North America [46, 77, 88, 97] and Europe [125, 131] were generally observed. All studies examining recent or lifetime cannabis use found increased risks with longer residence [88, 125, 131]. Studies on other drugs had mixed findings [46, 97, 131]. All [77, 88, 131] but one study [64] with a native reference suggested a convergence of immigrant drug use to native patterns, in some cases even surpassing native risks [131]. Findings were insufficient for meta-analysis.

### Substance Use Diagnoses and Dependence

All studies examining substance use diagnoses or dependence, including drug-related, alcohol-related or unspecified diagnoses, came from North America [29, 37, 38, 41, 42, 91, 92, 118, 126, 132]. Most studies found increased risks of substance use diagnosis with longer residence, for immigrants of all origins [41, 118, 126], and specifically for Asian [41, 132], African/Afro-Caribbean [91, 92] and Latino/Hispanic immigrants [29, 38], with only a few studies finding no change [37] or unclear trends in diagnosis [38, 132]. Most studies with a native reference indicated convergence to native risks of substance use diagnosis [29, 38, 41, 42, 92, 126, 132]. Findings were insufficient for meta-analysis.

### Physical Inactivity

Physical inactivity was divided into general/unspecified, leisure-time (e.g., exercise) and non-leisure-time (e.g., work-, travel-, household-related) physical inactivity. Studies examining indicators of physical activity were reverse interpreted.

North American evidence on changes in general physical inactivity by duration of residence varied from increased [98, 110, 113, 119, 127, 133–135, 142] to decreased [34, 51, 53, 54, 59, 62, 78, 115, 148] risks. Two European studies suggested either no change or increased inactivity with time [87, 108]. Studies from Australia, predominantly examining Asian or non-English-speaking immigrants, showed no consensus in the direction of behavioural changes [43, 76, 79, 137].

Meanwhile, immigrants largely decreased their risk of being inactive during leisure time with longer residence in North America, in both joint and sex-stratified analyses, as well as across multiple origins [27, 33, 44, 49, 54, 55, 59, 60, 63, 83, 89, 99, 110, 138, 144, 146, 148], excepting a few Canadian studies suggesting increased inactivity [93, 104, 109, 138]. European [56, 57, 108] and Australian [94] were limited and inconclusive. Non-leisure-time physical inactivity appeared to increase with longer residence in North America [27, 33, 59], especially among Latino/Hispanic and non-English-speaking immigrants [62, 68, 110, 148], with some conflicting evidence [54, 62, 148].

Altogether, most North American studies with native-born references suggested a convergence of physical inactivity patterns [34, 59, 60, 83, 109, 110, 113, 119, 138, 148], with only a few suggesting divergence [59, 84, 93, 138, 148]. Studies from outside North America showed mixed results regarding the direction of changes relative to natives.

**FIGURE 4:**
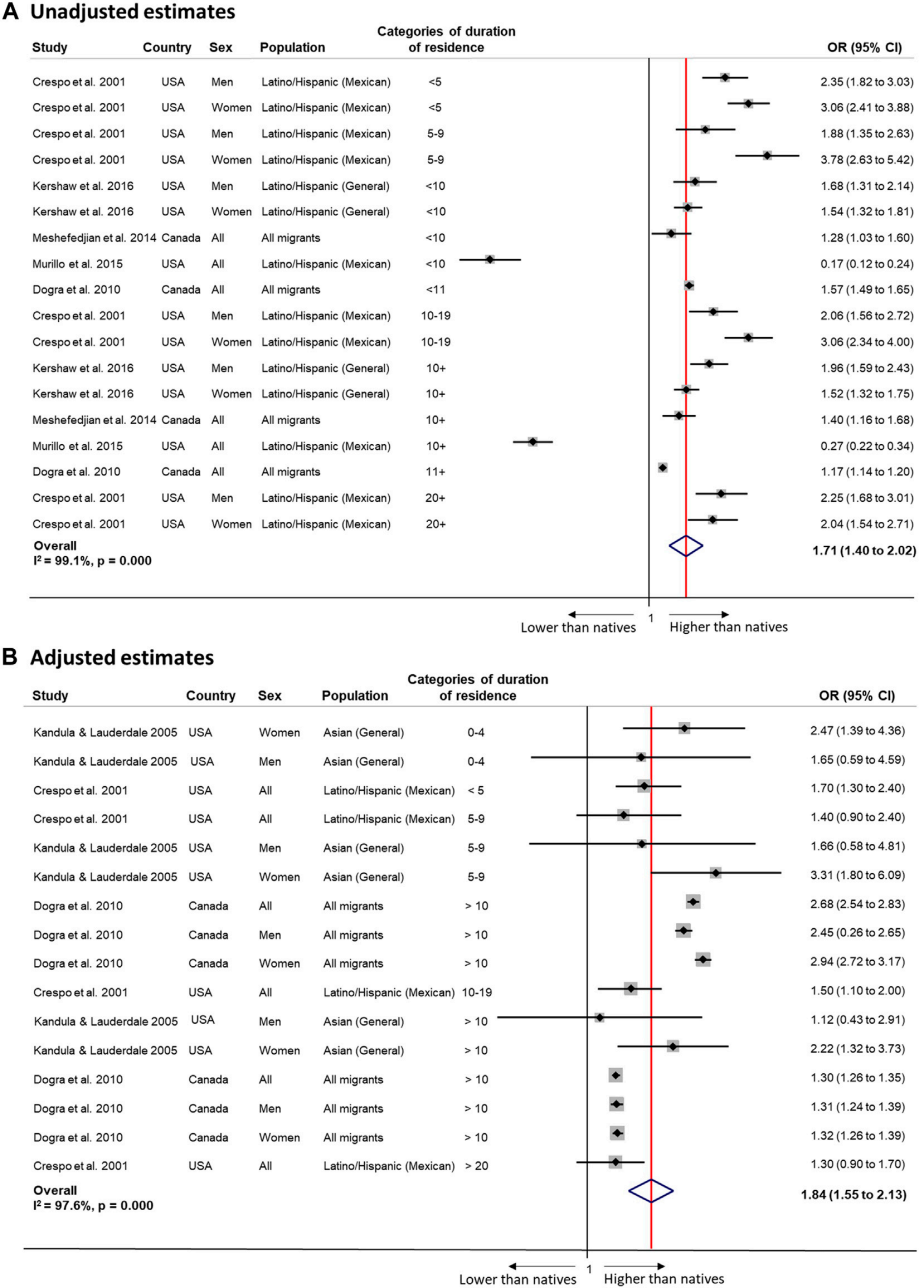
Random-effects meta-analysis of unadjusted (Panel **(A)**) and adjusted (Panel **(B)**) estimates of the association between physical activity and duration of residence among migrants compared to the native population (Sweden, 2022). Panel **(A)**: <5 years 2.67 (1.98 to 3.37), I^2^ = 53.2%, *p* 0.144 (2 estimates); ≥5 years 1.79 (1.38 to 2.20); I^2^ = 98.8%, *p* 0.000 (11 estimates); <10 years 1.88 (1.10 to 2.65); I^2^ = 98.0%, *p* 0.000 (8 estimates); ≥10 years 1.68 (1.23 to 2.10); I^2^ = 99.0%, *p* 0.000 (9 estimates); <15 years 1.66 (1.33 to 1.99); I^2^ = 99.2%, *p* 0.000 (16 estimates); ≥15 years 2.13 (1.69 to 2.57); I^2^ = 00.0%, *p* 0.645 (2 estimates). Panel **(B)**: <5 years 1.78 (1.29 to 2.28), I^2^ = 0.0%, *p* 0.629 (3 estimates); ≥5 years 1.83 (1.52 to 2.15); I^2^ = 98.1%, *p* 0.000 (13 estimates); <10 years 1.72 (1.32 to 2.12); I^2^ = 0.0%, *p* 0.578 (6 estimates); ≥10 years 1.31 (1 28 to 1 34); I^2^ = 0.0%, *p* 0.916 (5 estimates).

Meta-analyses showed higher odds of any physical inactivity among immigrants compared to natives with unadjusted (OR 1.71, 95% CI 1.40–2.02, *I* ^2^ = 99.1%) or adjusted estimates (OR 1.84, 95% CI 1.55–2.13, *I* ^2^ = 97.6%), but no patterns by duration of residence (Figure 4).

### Dietary Habits

Narrative synthesis of dietary changes by duration of residence were subdivided into summative measures of daily energy intake [33, 44, 66, 93–95, 105, 133, 139, 140] and various healthy eating indices [44, 58, 73, 84, 105, 111, 119, 130, 141]. North American studies found greater energy intake among various immigrant groups by longer residence [33, 44, 93], with some studies suggesting no change [139, 140] or even decreased [133] intake among Asian immigrants. European [66, 105], Australian [94] and Israeli [95] findings suggested increased levels of energy intake with longer residence. Regarding healthy eating indices, most North American studies found greater scores, i.e., less unhealthy diets, by longer residence for immigrants of African/Afro-Caribbean [44, 73] and Latino/Hispanic origin [84, 105, 119, 141]. Evidence from Europe was mixed [58, 105, 111]. Studies with native references found converging risks in Canada [93], but diverging risks among Latino/Hispanic immigrants in the US, relative to US-born Latino/Hispanics [84, 119]. Findings were insufficient for meta-analysis.

## Discussion

This systematic evaluation of the international literature demonstrates evidence of general changes in health risk behaviours by immigrants’ duration of residence, while also highlighting important heterogeneity in these patterns.

Narrative synthesis of the literature revealed that among general immigrant populations, especially women, increased risks of smoking were seen with longer residence in both North America and Europe. Similarly, North American studies demonstrated increased risks of (mild-to-moderate) alcohol and drug use with longer residence. Studies suggested increased levels of energy intake and less unhealthy dietary patterns, with decreased leisure-time but increased non-leisure-time inactivity risks with longer residence in North America. There were no consistent patterns in European studies for alcohol use, physical inactivity or diet scores. Although the narrative synthesis indicated that smoking, alcohol and drug use, and physical inactivity risks tended to converge to native-born reference levels in North America, studies of convergence or divergence in other contexts (mainly Europe) were fewer and less conclusive. The results of our narrative synthesis call for consideration of duration of residence as an instrument to monitor immigrants’ health behaviours over time in the receiving country.

Overall, the meta-analyses showed lower odds of smoking and alcohol use, but higher odds of physical inactivity among immigrants compared to natives, offering evidence of systematic differences in health risk behaviours between immigrants and natives. Yet, despite some signs of convergence, i.e., for alcohol use in North America, the meta-analyses did not provide robust support for a universal trend in health behaviours by duration of residence, nor for the convergence of risk behaviours to native levels (i.e., “regression to the mean”). The lack of such a universal pattern illustrates the role of different factors in immigrants’ adoption of health risk behaviours, contingent on the heterogeneity existing between specific migrant groups and within the reference native population. This observation deviates to a large extent from other universal trends (e.g., the healthy immigrant paradox) [24] which have been corroborated by meta-analyses despite large heterogeneity between groups.

Given that this is the first systematic review to consider immigrants’ changes in health behaviours by duration of residence, the reason for the lack of a universal trend across country contexts remains unknown. Previous studies considering health behavioural changes by acculturation (rather than duration of residence) attribute this heterogeneity to differences in the prevalence of health risk behaviours in the country of origin and destination, in combination with differences in origin and destination country norms and values (i.e., the operant model of acculturation) [149, 150]. The complexity of our review—which considers international evidence for many immigrant populations, receiving country contexts and health risk behaviours across a long time-span—does not permit a close evaluation of any specific model of change. However, we believe that our study offers a solid basis to build further studies in this direction.

Several important theoretical limitations with implications for public health interventions were identified in this review. Most studies assessing changes by duration of residence (alone or in combination with acculturation indices) motivate or interpret their results as a process of cultural assimilation (i.e., acculturation). The hegemonic use of cultural arguments to motivate the study of duration of residence effects lacks empirical support and is not sufficiently justified from a theoretical point of view. Most importantly, this practice could limit consideration of alternative, time-dependent mechanisms and potential targets for public health intervention. Cumulative experiences of (increasing) social inequalities in the receiving country could alternatively explain changes in health risk behaviours. For instance, lack of material resources could limit possibilities to make healthy choices, including buying healthy food or participating in physical activity, while unhealthy habits could further stem from the psychosocial stress of economic instability as well as racism or discrimination [151–153]. Thus, the social determinants of health [154] may be more relevant for the interpretation of these results than acculturation, with social policies as important instruments of change. Within this framework, our lack of evidence for a universal pattern of change in health risk behaviours could reflect heterogeneity stemming from various upstream social factors in the receiving country. In fact, recent systematic evidence has highlighted the often uncaptured adverse health effects of restrictive entry and integration policies for immigrants worldwide [155].

Our efforts to synthesise the literature also revealed several methodological limitations. Importantly, most of the captured studies used cross-sectional data, limiting the evaluation and interpretation of actual behavioural changes over time. Although studies include multiple controls (e.g., education, employment, income, marital status, language and acculturation), authors may unwittingly control for potential mediators (i.e., explanations for differences) in their effort to rule out compositional differences between groups with different lengths of residence. This practice is common and even less justified in longitudinal studies. For this reason, we ran meta-analyses using available unadjusted estimates (or when data allowed for calculation) to be able to evaluate possible statistical bias. However, models using unadjusted estimates led to similar conclusions, so it is unlikely that the lack of concrete findings regarding health-behavioural patterning is due to over-adjustment.

Certain reference groups, i.e., established immigrants, may also have enlarged compositional differences, making it difficult to test convergence to the norm. Furthermore, these populations may represent a selectively healthy group, given the increasing propensity of return migration with poorer health (i.e., so-called salmon bias) [156]. Thus, we focused on native reference populations in the meta-analyses. In some studies, second-generation immigrants were also equated as natives, and although not immigrants themselves, the second generation may continue to experience stable or growing health inequalities. Including them as a reference population could lead to over- or under-estimation of first-generation changes.

The lack of justification for different duration of residence categories is also a limitation. Studies typically did not specify whether this selection was data-driven, dependent on receiving-country-specific migration trajectories or corresponding to international conventions. Researchers should develop a standardised operationalisation of duration of residence categories with public health relevance to facilitate international comparisons, e.g., by five-, ten- or fifteen-year intervals.

Data limitations, such as small sample size, might have constrained the possibility of evaluating variations by country of birth when assessing changes in immigrants’ health risk behaviours, as well as the possibility of offering gender-specific comparisons. This was especially the case for the meta-analysed studies, which represented a subset of the published studies on the topic, thus limiting the generalisability of the meta-analysis findings beyond that of the narrative synthesis results. Overall, further studies are needed to fill this gap as well as to contribute evidence from other areas of the world, beyond North America and Europe.

Given that research is more likely to exclude insignificant duration of residence effects in favour of general associations between migration status and health risk behaviours, any publication bias would likely have led to an overestimation of duration of residence effects, which we do not observe. Yet, given that only studies with duration of residence measures were included, our results of overall health behavioural differences between immigrants and natives, irrespective of duration of residence, should be interpreted with caution. On the other hand, these results could be more representative of immigrant populations than results from single studies which do not consider duration of residence, as the latter may overrepresent newly-arrived or long-term immigrants.

To our knowledge, this is the first study to comprehensively synthesise 20 years of international evidence on changes in health risk behaviours by immigrants’ duration of residence. We found evidence for changing patterns by immigrants’ duration of residence, yet the existence of a clear, universal trend in behavioural changes was called into question by the meta-analyses. This nuance highlights the importance of considering alternative theoretical frameworks regarding the role of receiving country contexts, including structural factors as well as entry and integration policies, as potential explanations of this variation. Finally, this evaluation identified several methodological challenges to address in future research, including the need for longitudinal studies, comparative duration of residence categorisations, deliberate adjustment methods and thorough consideration of reference populations.

### Conclusion

Duration of residence could serve as an effective public health instrument for monitoring, predicting and preventing developments in immigrants’ health risk behaviours, conditional on immigrants’ background characteristics and contextual factors. In combination with other relevant information, such as age at migration and country of origin, this instrument could help to tailor and evaluate targeted public health interventions by considering when (with regards to time after migration) to act for specific immigrant populations in specific contexts. Duration of residence data can be easily collected through questionnaires and administrative registers. Furthermore, it is increasingly acknowledged that the adoption of health risk behaviours is largely influenced by socioeconomic conditions at the individual, community and societal levels, factors which cannot be captured by traditional acculturation measures but can be operationalised within the duration of residence framework. Thus, by studying patterns of health risk behaviours by immigrants’ duration of residence in different receiving contexts, researchers can evaluate the role of societal conditions in shaping immigrant health over time.
