# Supplementary material for: Health Risk Behaviours by Immigrants’ Duration of Residence: A Systematic Review and Meta-Analysis
Source: Int J Public Health. 2022 Aug 5;67:1604437. doi: 10.3389/ijph.2022.1604437 (PMC9388735; doi:10.3389/ijph.2022.1604437)
Supplement: Supplementary file 1 [file DataSheet1.docx]

**Supplementary material. Health risk behaviors by immigrants’ duration of residence: a systematic review and meta-analysis**

**Table 1.** Electronic search strategy: Example

| **Database:** PubMed/MEDLINE |
| --- |
| **Publication dates:** January 1^st^ 2000 - December 31^st^ 2019 |
| **Other limits:** None |
| **Search string:** (((Emigrants and Immigrants[Mesh]) OR (Emigration and Immigration[Mesh]) OR (Refugees[Mesh]) OR (Transients and Migrants[Mesh]) OR (Ethnic Groups[Mesh])) OR  ((asylum*[Title/Abstract]) OR (country of birth[Title/Abstract]) OR (countries of birth[Title/Abstract]) OR (country of origin[Title/Abstract]) OR (countries of origin[Title/Abstract]) OR (displaced individual*[Title/Abstract]) OR (displaced people*[Title/Abstract]) OR (displaced person*[Title/Abstract]) OR (displaced population*[Title/Abstract]) OR (displaced women*[Title/Abstract]) OR (emigra*[Title/Abstract]) OR (ethnic*[Title/Abstract]) OR (expat*[Title/Abstract]) OR (evacuat*[Title/Abstract]) OR (evacuee*[Title/Abstract]) OR (foreign background*[Title/Abstract]) OR (foreign-born[Title/Abstract]) OR (foreign born[Title/Abstract]) OR (foreigner*[Title/Abstract]) OR (immigra*[Title/Abstract]) OR (migrant*[Title/Abstract]) OR (migrate*[Title/Abstract]) OR (migration*[Title/Abstract]) OR (refugee*[Title/Abstract])))  **AND**  (((Acculturation[Mesh])) OR  ((accultur*[Title/Abstract]) OR (assimilat*[Title/Abstract]) OR (duration of residenc*[Title/Abstract]) OR (duration of stay[Title/Abstract]) OR (length of residenc*[Title/Abstract]) OR (length of stay[Title/Abstract]) OR (length of time[Title/Abstract]) OR (years of residenc*[Title/Abstract]) OR (years of stay*[Title/Abstract]) OR (long time*[Title/Abstract]) OR (longer time*[Title/Abstract]) OR (long stay*[Title/Abstract]) OR (longer stay*[Title/Abstract]) OR (short time*[Title/Abstract]) OR (shorter time*[Title/Abstract]) OR (short stay*[Title/Abstract]) OR (shorter stay*[Title/Abstract]) OR (recent immigr*[Title/Abstract]) OR (recently immigr*[Title/Abstract])))  **AND**  (((Health Risk Behaviors[Mesh]) OR (Risk-Taking[Mesh]) OR (Substance-Related Disorders[Mesh]) OR (Drug-Seeking Behavior[Mesh]) OR (Drug Misuse[Mesh]) OR (Alcohol Drinking[Mesh]) OR (Alcoholism[Mesh]) OR (Alcoholic Beverages[Mesh]) OR (Drinking Behavior[Mesh]) OR (Adolescent Behavior[Mesh]) OR (Smoking[Mesh]) OR (Tobacco Use[Mesh]) OR (Smoking Cessation[Mesh]) OR (Tobacco Use Cessation[Mesh]) OR (Cannabis[Mesh]) OR (Marijuana Use[Mesh]) OR (Narcotics[Mesh]) OR (Morphine Derivatives[Mesh]) OR (Amphetamine[Mesh]) OR (Cocaine[Mesh])) OR  ((risk behavio*[Title/Abstract]) OR (risky behavio*[Title/Abstract]) OR (risk taking*[Title/Abstract]) OR (risk-taking[Title/Abstract]) OR (addict*[Title/Abstract]) OR (dependen*[Title/Abstract]) OR (alcohol*[Title/Abstract]) OR (drink*[Title/Abstract]) OR (bing*[Title/Abstract]) OR (spirits*[Title/Abstract]) OR (liquor*[Title/Abstract]) OR (wine*[Title/Abstract]) OR (beer*[Title/Abstract]) OR (smok*[Title/Abstract]) OR (nicotine*[Title/Abstract]) OR (tobacco*[Title/Abstract]) OR (cigarette*[Title/Abstract]) OR (marijuana*[Title/Abstract]) OR (cannabis*[Title/Abstract]) OR (hashish*[Title/Abstract]) OR (snus*[Title/Abstract]) OR (snuff*[Title/Abstract]) OR (substance*[Title/Abstract]) OR (drug*[Title/Abstract]) OR (opioid*[Title/Abstract]) OR (opiate*[Title/Abstract]) OR (narcotic*[Title/Abstract]) OR (amphetamine*[Title/Abstract]) OR (cocain*[Title/Abstract]) OR (LSD[Title/Abstract]) OR (ecstasy[Title/Abstract]) OR (hallucinogen*[Title/Abstract]) OR (psilocybin[Title/Abstract]) OR (physical activit*[Title/Abstract]) OR (physically active[Title/Abstract]) OR (fitness*[Title/Abstract]) OR (workout*[Title/Abstract]) OR (exercis*[Title/Abstract]) OR (diet[Title/Abstract]) OR (diets[Title/Abstract]) OR (dietary[Title/Abstract]) OR (dieting[Title/Abstract]) OR (nutrition*[Title/Abstract]) OR (eating*[Title/Abstract]))) |

**Table 2.** Descriptive overview of the 123 studies included in the systematic review

| **Ref.** | **Authors, Year** | **Country** | **Data source(s)** | **Study design** | **Data years** | **Follow-up period** | **Migrant population(s)** | **Sex** | **Duration of residence measure** | **Health risk behavior(s)** |
| --- | --- | --- | --- | --- | --- | --- | --- | --- | --- | --- |
| 15 | Addo et al. 2018 | Netherlands  Germany  UK | Research on Obesity & Diabetes among African Migrants (RODAM) Study | Cross-sectional | 2012-15 | N/A | African (Ghanaian) | Stratified | *Categorical*  1-5 years  5-9 years  10+ years (Ref.) | Alcohol use |
| 16 | Afable et al. 2016 | USA  (New York) | Chinese American Cardiovascular Health Assessment (CHA CHA) | Cross-sectional | 2010-11 | N/A | Asian (Chinese) | All | *Categorical*  <6 years (Ref.)  6-15 years  15+ years | Physical inactivity |
| 17 | Agic et al. 2016 | Canada  (Ontario) | Centre for Addiction & Mental Health (CAMH) Monitor | Cross-sectional | 2005-10 | N/A | All (*unspecified*) | All | *Categorical*  <6 years  6-10 years  11-20 years  >20 years | Alcohol use |
| 18 | Alegria et al. 2007 | USA | National Latino & Asian American Study (NLAAS) | Cross-sectional | 2002-03 | N/A | Latino/Hispanic | Men | *Categorical*  <6 years  6-10 years  11-20 years  20+ years  Native-born (Ref.) | Substance use |
| 19 | Amundsen 2012 | Norway | Oslo Immigrant Health Study (HUBRO) | Cross-sectional | 2000-01; 2002 | N/A | Middle Eastern (Iranian, Turkish, Pakistani) | All | *Continuous* | Alcohol use |
| 20 | An et al. 2008 | USA  (California) | California Health Interview Survey (CHIS) | Pooled cross-sectional | 2001, 2003 | N/A | Asian | Stratified | *Categorical*  <15 years (Ref.)  15+ years | Smoking |
| 21 | Azagba et al. 2019 | USA | Current Population Survey - Tobacco Use Supplement (CPS-TUS) | Pooled cross-sectional | 2014-15 | N/A | All (*unspecified*) | All / Stratified | *Categorical*  <6 years  6-10 years  11-20 years  20+ years  Native-born (Ref.) | Smoking |
| 22 | Basu & Insler 2019 | USA | National Health & Nutrition Examination Survey (NHANES) | Pooled cross-sectional | 4 waves: 2003-04 to  2009-10 | N/A | All (*unspecified*) | All | *Continuous* | Physical inactivity;  Diet |
| 23 | Bayog & Waters 2018 | USA  (California) | CHIS [6] | Cross-sectional | 2011-12 | N/A | Asian (Filipino) | All | *Categorical*  <15 years  15+ years  Native-born (Ref.) | Smoking; Physical inactivity |
| 24 | Bharmal et al. 2015 | USA  (California) | CHIS [6] | Pooled cross-sectional | 2005, 2007, 2009 | N/A | Asian (Southern) | All | *Categorical*  <5 years  5-10 years  10-15 years  15+ years (Ref.) | Alcohol use; Physical inactivity |
| 25 | Bingham et al. 2016 | USA | Africans in America Cohort | Cross-sectional | *Not reported* | N/A | African | All | *Categorical*  <10 years (Ref.)  10+ years | Smoking; Alcohol use;  Physical inactivity |
| 26 | Blanco et al. 2013 | USA | National Epidemiologic Survey on Alcohol & Related Conditions (NESARC) | Cross-sectional | 2004-05 | N/A | Latino/Hispanic | All | *Categorical*  <18 years  18+ years  Native-born (Ref.) | Substance use |
| 27 | Borges et al. 2006 | USA | US National Alcohol Survey (NAS) | Pooled cross-sectional | 1995, 2000 | N/A | Latino/Hispanic (Mexican) | All / Stratified | *Categorical*  <7 years (Ref.)  7-12 years  13-20 years  21-55 years  Native-born | Alcohol use |
| 28 | Bostean et al. 2017 | USA | National Health Interview Survey (NHIS) | Pooled cross-sectional / Cohort | 1998, 2003, 2008, 2013 | 15 years (5-year segments) | Latino/Hispanic | Stratified | *Categorical*  <5 years  5-9 years  10-14 years  Native-born (Ref.) | Smoking |
| 29 | Brathwaite et al. 2017 | Netherlands  Germany  UK | RODAM [1] | Cross-sectional | 2012-15 | N/A | African (Ghanaian) | All | *Categorical*  <10 years (Ref.)  10-19 years  20-29 years  30+ years | Smoking |
| 30 | Breslau et al. 2006 | USA | NESARC [12] | Cross-sectional | 2000-01 | N/A | Asian | All | *Categorical*  <6 years  6-15 years  16+ years  Native-born (Ref.) | Substance use |
| 31 | Breslau et al. 2007 | USA | National Comorbidity Survey Replication (NCS-R) | Cross-sectional | 2001-03 | N/A | All (*unspecified*) | All | *Categorical*  <6 years  6-10 years  11+ years  Native-born (Ref.) | Substance use |
| 32 | Brock et al. 2001 | Australia  (New South Wales) | *Unnamed study (survey)* | Cross-sectional | 1999 | N/A | Asian (Vietnamese) | All /  Men | *Categorical*  <13 years (Ref.)  13+ years | Smoking;  Alcohol use;  Physical inactivity |
| 33 | Brown et al. 2018 | USA | NHANES [8] | Pooled cross-sectional | 2003-12 | N/A | African/Afro-Caribbean (Non-Latino Black) | All | *Categorical*  <10 years (Ref.)  10-19 years  20-29 years  30+ years | Smoking;  Physical inactivity;  Diet |
| 34 | Canfield et al. 2017 | UK  (London) | *Unnamed study (survey)* | Cross-sectional | 2013 | N/A | Latino/Hispanic (Brazilian) | All | *Continuous* | Smoking;  Alcohol use;  Drug use |
| 35 | Cano et al. 2017 | USA  (Florida) | *Unnamed study (survey)* | Cross-sectional | *Not reported* | N/A | Latino/Hispanic | All | *Continuous* | Alcohol use |
| 36 | Castañeda et al. 2019 | USA  (Illinois, Florida, New York, California) | Hispanic Community Health Study/Study of Latinos (HCHS/SOL) | Cross-sectional | 2008-11 | N/A | Latino/Hispanic | Stratified | *Categorical*  <10 years  10+ years  Native-born (Ref.) | Alcohol use |
| 37 | Chartier et al. 2017 | USA | NESARC [12] | Longitudinal | Wave 1:  2001-02; Wave 2:  2004-05 | 3 years | Latino/Hispanic | All | *Continuous* | Alcohol use |
| 38 | Choi et al. 2008 | USA | *Unnamed study (survey)* | Cross-sectional | 2003-04 | N/A | Asian (Korean) | Women | *Continuous* | Physical inactivity |
| 39 | Chou et al. 2010 | USA | NHIS [14] | Cross-sectional | 1998-2004 | N/A | Asian (Chinese) | All | *Categorical*  <5 years (Ref.)  5-14 years  15+ years | Smoking |
| 40 | Commodore-Mensah et al. 2018 | USA  (Maryland, Washington DC) | Afro-Cardiac Study | Cross-sectional | 2013-14 | N/A | African (Ghanaian, Nigerian) | Stratified | *Categorical*  <10 years  10+ years (Ref.) | Physical inactivity |
| 41 | Constantine et al. 2013 | USA  (Minnesota) | Diverse Racial Ethnic Groups & Nations (DREGAN) Study | Cross-sectional | 2006-07 | N/A | Asian (Southeastern);  Latino/Hispanic | Stratified | *Continuous* | Smoking |
| 42 | Coronado et al. 2008 | USA  (Washington) | *Unnamed study (survey)* | Cross-sectional | 2006-07 | N/A | Asian (Vietnamese) | Women | *Categorical*  <10 years (Ref.)  10-19 years  20+ years | Smoking;  Physical inactivity |
| 43 | Creighton et al. 2012 | USA  (California) | Los Angeles Family and Neighborhood Survey (L.A. FANS) | Cross-sectional | Wave 2:  2006-08 | N/A | Latino/Hispanic (Mexican) | All | *Categorical*  <16 years (Ref.)  16+ years | Physical inactivity |
| 44 | Crespo et al. 2001 | USA | NHANES [8] | Cross-sectional | 1988-1994 | N/A | Latino/Hispanic (Mexican) | All | *Categorical*  <5 years  5-9 years  10-19 years  20+ years  Native-born (Ref.) | Physical inactivity |
| 45 | Dawson et al. 2005 | Sweden | Swedish Survey of Living Conditions (ULF-SILC) | Pooled cross-sectional | 1996, 1997, 1999 | N/A | All (*unspecified*) | Stratified | *Categorical*  <5 years  5-9 years  10-14 years  15-19 years  20+ years (Ref.) | Physical inactivity |
| 46 | de Munter et al. 2013 | Netherlands UK | Surinamese in the Netherlands: Study on Ethnicity and Health (SUNSET; Netherlands); Health Survey (UK) | Pooled cross-sectional | SUNSET:  2001-03; Health Survey:  1998, 1999, 2003, 2004 | N/A | Asian (Surinamese);  African/Afro-Caribbean (Surinamese) | All | *Categorical*  <24 years  24-33 years  >33 years  Native-born (Ref.) | Physical inactivity |
| 47 | Delisle et al. 2009 | Spain | *Unnamed study (survey)* | Cross-sectional | 2001 | N/A | African (Western) | All | *Categorical*  <6 years (Ref.)  6-10 years  11+ years | Diet |
| 48 | Dogra et al. 2010 | Canada | Canadian Community Health Survey (CCHS) | Pooled cross-sectional | 2000-05 | N/A | All (*unspecified*) | All / Stratified | *Categorical*  <11 years  11+ years  Native-born (Ref.) | Physical inactivity |
| 49 | Echeverria et al. 2013 | USA | NHIS [14] | Cross-sectional | 2010 | N/A | Latino/Hispanic | All | *Categorical*  <10 years  10+ years  Native-born (Ref.) | Smoking; Physical inactivity |
| 50 | Ertl et al. 2018 | USA  (Florida) | *Unnamed study* | Cross-sectional | *Not reported* | N/A | Latina/Hispanic | Women | *Continuous* | Alcohol use |
| 51 | Evenson et al. 2004 | USA  (North Carolina) | *Unnamed study (interview)* | Cross-sectional | 2001-02 | N/A | Latina/Hispanic | Women | *Categorical*  <3 years (Ref.)  3+ years | Physical inactivity |
| 52 | Gaskins et al. 2012 | USA | NHANES [8] | Pooled cross-sectional | 1988-94 | N/A | All (*unspecified*) | Women | *Continuous* | Physical inactivity |
| 53 | Gazard et al. 2015 | UK | South East London Community Health (SELCoH) Study | Cross-sectional | 2008-10 | N/A | All (*unspecified*) | All | *Categorical*  <5 years  5-10 years  10+ years  Native-born (Ref.) | Smoking;  Alcohol use;  Drug use |
| 54 | Glenn et al. 2009 | USA  (California) | South Asian Network (SAN) Needs Assessment | Cross-sectional | 2004-05 | N/A | Asian (Southern) | Men | *Continuous* | Smoking |
| 55 | Goff et al. 2015 | UK | *Unnamed study (survey)* | Cross-sectional | 2011-12 | N/A | African/Afro-Caribbean | All | *Categorical*  <13 years  13-38 years  >38 years | Diet |
| 56 | Gorman et al. 2014 | USA | NLAAS [4] | Cross-sectional | 2002-03 | N/A | Asian;  Latino/Hispanic | All | *Categorical*  <5 years (Ref.)  5-10 years  11-20 years  21+ years | Smoking |
| 57 | Ham et al. 2007 | USA | National Household Travel Survey (NHTS); NHIS [14] - Cancer Supplement (NHIS-CS); NHANES [8] | Pooled cross-sectional | NHTS: 2001-02; NHIS-CS: 2000; NHANES:  1999-2002 | N/A | Latino/Hispanic | All | *Categorical*  <5 years  5-9 years  10-14 years  15+ years | Physical inactivity |
| 58 | Hardie et al. 2013 | USA | NHIS [14] | Cross-sectional | 2009 | N/A | Latino/Hispanic (Mexican) | Women | *Continuous* | Alcohol use |
| 59 | Hislop et al. 2008 | Canada | *Unnamed study* | Cross-sectional | 2005 | N/A | Asian (Chinese) | Men | *Categorical*  <10 years  10+ years | Smoking;  Physical inactivity |
| 60 | Holmes & Marcelli 2014 | USA  (Massachusetts) | 2007 Harvard-UMASS Boston Metropolitan Immigrant Health and Legal Status Survey (BM-IHLSS) | Cross-sectional | 2007 | N/A | Latino/Hispanic (Brazilian) | All | *Continuous* | Smoking |
| 61 | Hu et al. 2010 | USA  (Texas) | *Unnamed study*  *(survey/interview)* | Cross-sectional | 2004 | N/A | Asian (Chinese) | All | *Categorical*  <6 years  6-10 years  11-15 years  >15 years (Ref.) | Smoking |
| 62 | Huffman et al. 2014 | USA | *Unnamed study (interviews)* | Cross-sectional | *Not reported* | N/A | Afro-Caribbean (Haitian) | All | *Continuous* | Diet |
| 63 | Hyman et al. 2008 | Canada  (Ontario) | *Unnamed study (structured interviews)* | Cross-sectional | 1999-2000 | N/A | African (Ethiopian) | Stratified | *Continuous* | Smoking |
| 64 | Jayaweera & Quigley 2010 | UK | The Millennium Cohort Study (MCS) | Cross-sectional | Wave 1:  2001-02 | N/A | All (*unspecified*) | Women | *Categorical*  <3 years (Ref.)  4-6 years  7-9 years  10+ years | Smoking;  Alcohol use |
| 65 | Jin et al. 2017 | Australia | The 45 and Up Study | Cross-sectional | 2006-09 | N/A | Asian (Chinese) | Stratified | *Categorical*  <10 years (Ref.)  10-19 years  20-29 years  30+ years | Smoking; Physical inactivity |
| 66 | Johnson et al. 2002 | USA | NHIS [14] Drug and Alcohol Use Data File (NHIS-DAU) | Cross-sectional | 1991 | N/A | All (*unspecified*) | All | *Categorical*  <1 year  1-4 years  5-9 years  10-14 years  15+ years (Ref.) | Alcohol use;  Drug use |
| 67 | Jonnalagadda & Diwan 2005 | USA | *Unnamed study (survey)* | Cross-sectional | *Not reported* | N/A | Asian (Indian) | All | *Continuous* | Smoking;  Physical inactivity |
| 68 | Joshi et al. 2017 | Australia | Household, Income and Labor Dynamics in Australia (HILDA) | Longitudinal | Waves 1-12:  2001-12 | 12 years | All (*unspecified*) | All | *Categorical*  <10 years  10-19 years  20+ years  Native-born (Ref.) | Physical inactivity |
| 69 | Joshi et al. 2018 | Australia | HILDA [54] | Longitudinal | Waves 1-12:  2001-12 | 12 years | All (*unspecified*) | All | *Categorical*  <10 years  10-19 years  20+ years  Native-born (Ref.) | Smoking |
| 70 | Juon et al. 2003 | USA  (Maryland) | Healthy Korean American Project | Cross-sectional | 1998-99 | N/A | Asian (Korean) | Men | *Categorical*  <10 years (Ref.)  10-19 years  20+ years | Smoking |
| 71 | Kabir et al. 2008 | Ireland | *Unnamed study (survey)* | Cross-sectional | 2007 | N/A | European (Polish) | All | *Categorical*  <1 year  1-2 years  >2 years (Ref.) | Smoking |
| 72 | Kandula & Lauderdale 2005 | USA | CHIS [6] | Cross-sectional | 2001 | N/A | Asian | Stratified | *Categorical*  <5 years  5-9 years  10+ years  Native-born (Ref.) | Physical inactivity |
| 73 | Kershaw et al. 2016 | USA | HCHS/SOL [22] | Cross-sectional | 2008-11 | N/A | Latino/Hispanic | Stratified | *Categorical*  <10 years  10+ years  Native-born (Ref.) | Physical inactivity;  Diet |
| 74 | Khlat et al. 2019 | France | The 2010 ‘Health Barometer’ | Longitudinal (retrospective) | 2010 | Up to 60 years | African (Maghrebian,  Sub-Saharan) | Stratified | *Categorical*  <6 years  6-9 years  10+ years  Native-born (Ref.) | Smoking |
| 75 | Kim et al. 2014 | USA | *Unnamed study (survey)* | Cross-sectional | *Not reported* | N/A | Asian | Men | *Continuous* | Alcohol use |
| 76 | Koca & Lapa 2014 | Germany  UK | *Unnamed study* | Cross-sectional | 2011-12 | N/A | Middle Eastern (Turkish) | All | *Categorical*  Germany:  <26 years  26+ years (Ref.)  UK:  <16 years  16+ years (Ref.) | Physical inactivity |
| 77 | Kolar et al. 2018 | Canada | *Unnamed study (survey)* | Cross-sectional | 2011-12 | N/A | All (*unspecified; university students*) | All | *Categorical*  <5 years  5+ years  Native-born (Ref.) | Drug use |
| 78 | Koya & Egede 2007 | USA | NHIS [14] | Cross-sectional | 2002 | N/A | All (*unspecified*) | All / Stratified | *Categorical*  <10 years (Ref.)  10-14 years  15+ years | Smoking;  Physical inactivity |
| 79 | Kuerban 2016 | USA | CPS-TUS [7] | Pooled cross-sectional | 1998-99; 2005-06; 2010-11 | N/A | Asian | All | *Categorical*  <10 years (Ref.)  10-20 years  20-30 years  30+ years | Smoking |
| 80 | Lacey et al. 2015 | USA | National Survey of American Life (NSAL) | Cross-sectional | 2001-03 | N/A | Afro-Caribbean | All | *Categorical*  <20 years (Ref.)  20+ years | Alcohol use;  Drug use;  Substance use |
| 81 | Lacey et al. 2016 | USA | NSAL [66] | Pooled cross-sectional | 2001-03 | N/A | Afro-Caribbean | All | *Categorical*  0-5 years  6-10 years  11-20 years  20+ years  Native-born (Ref.) | Substance use |
| 82 | Lear et al. 2009 | Canada | Multicultural Community Health Assessment Trial (M-CHAT) | Cross-sectional | *Not reported* | N/A | Asian (Chinese, Southern);  European | All | *Categorical*  <10 years  10-20 years  20-30 years  30+ years  Native-born (Ref.) | Smoking;  Physical inactivity;  Diet |
| 83 | Lee et al. 2007 | Australia  (New South Wales) | *Unnamed study (survey)* | Cross-sectional | *Not reported* | N/A | Asian (Taiwanese) | Women | *Categorical*  <5 years (Ref.)  5+ years | Physical inactivity;  Diet |
| 84 | Leshem & Dessie-Navon 2018 | Israel | *Unnamed study (survey)* | Cross-sectional | *Not reported* | N/A | African (Ethiopian) | All | Short (approx. 6 months) vs. long (approx. 3 years) | Smoking;  Physical inactivity; Diet |
| 85 | Leung 2014 | USA | CPS-TUS [7] | Pooled cross-sectional | 1995-2007 (17 waves) | N/A | All (*unspecified*) | All | *Continuous* | Smoking |
| 86 | Levy et al. 2005 | USA  (California) | HEY Man (Health Evaluation in Young Men) Study | Cross-sectional | 2001-03 | N/A | Latino/Hispanic | Men | *Categorical*  <5 years  5+ years | Drug use |
| 87 | Li & Hummer 2015 | USA | New Immigrant Survey (NIS) | Cross-sectional | Wave 1: 2003 | N/A | Asian | All | *Categorical*  <10 years (Ref.)  10+ years  *Continuous* | Smoking;  Physical inactivity |
| 88 | Li & Wen 2013 | USA | CHIS [6] | Cross-sectional | 2007 | N/A | All (*unspecified*) | All | *Continuous* | Physical inactivity |
| 89 | Li & Wen 2015 | USA | NIS [73] | Cross-sectional | Wave 1: 2003 | N/A | All (*unspecified*) | Stratified | *Continuous* | Smoking;  Alcohol use |
| 90 | Lo et al. 2014 | USA | NHIS [14] | Cross-sectional | 2008-11 | N/A | Asian | All | *Categorical*  <5 years  5+ years  Native-born (Ref.) | Alcohol use |
| 91 | Lopez-Gonzalez et al. 2005 | USA | NHIS [14] | Cross-sectional | 1998-2001 | N/A | All (*unspecified*) | All / Stratified | *Categorical*  <10 years  10+ years  Native-born (Ref.) | Smoking;  Alcohol use |
| 92 | Maffini et al. 2015 | USA | NESARC [12] | Cross-sectional | 2001-02 | N/A | Asian | All | *Continuous* | Smoking |
| 93 | Mahmood et al. 2019 | Canada | CCHS [34] | Cross-sectional | 2011-12 | N/A | All (*unspecified*) | All | *Categorical*  <10 years  10+ years (Ref.) | Physical inactivity |
| 94 | Marin-Guerrero et al. 2015 | Spain | Study on Nutrition and Cardiovascular Risk in Spain (ENRICA) Study | Cross-sectional | 2008-10 | N/A | Latino/Hispanic (Latin American) | All | *Categorical*  <5 years (Ref.)  5-9 years  10+ years | Diet |
| 95 | Maxwell et al. 2005 | USA  (California) | CHIS [6] | Cross-sectional | 2001 | N/A | Asian;  Latino/Hispanic | Stratified | *Categorical*  <15 years (Ref.)  15+ years | Smoking |
| 96 | Maxwell et al. 2007 | USA  (California) | *Unnamed study (survey)* | Cross-sectional | 2004-05 | N/A | Asian (Filipino) | Men | *Continuous* | Smoking |
| 97 | Mejean et al. 2009 | France | *Unnamed study (survey)* | Cross-sectional | 2004 | N/A | African (Tunisian) | Men | *Categorical*  <10 years  10-29 years  30+ years | Physical inactivity |
| 98 | Meshefedjian et al. 2014 | Canada  (Quebec) | CCHS [34] | Cross-sectional | 2009-10 | N/A | All (*unspecified*) | All | *Categorical*  <10 years  10+ years  Native-born (Ref.) | Smoking;  Alcohol use;  Physical inactivity |
| 99 | Murillo et al. 2015 | USA | NHANES [8] | Cross-sectional | 2007-10 | N/A | Latino/Hispanic (Mexican) | All | *Categorical*  <10 years  10+ years  Native-born (Ref.) | Physical inactivity |
| 100 | Nicolaou et al. 2006 | Netherlands | SUNSET [32] | Cross-sectional | 2001-03 | N/A | Asian (Surinamese);  Afro-Caribbean (Surinamese) | Stratified | *Continuous* | Diet |
| 101 | Osei-Kwasi et al. 2019 | Netherlands  Germany  UK | RODAM [1] | Cross-sectional | 2012-15 | N/A | African (Ghanaian) | All | *Categorical*  0-10 years (Ref.)  11-20 years  21+ years | Alcohol use |
| 102 | Osypuk et al. 2009 | USA | Multiethnic Study of Atherosclerosis (MESA) | Longitudinal | 2000-12 | 10 years | Asian (Chinese); Latino/Hispanic | All | *Categorical*  <15 years  15-30 years  30+ years  Native-born (Ref.) | Physical inactivity |
| 103 | Pampel et al. 2020 | USA | NHIS [14] | Pooled cross-sectional | 2000-15 | N/A | All (*unspecified*) | Stratified | *Categorical*  <1 year  1-4 years  5-9 years  10-14 years  15+ years  Native-born (Ref.) | Smoking |
| 104 | Parikh et al. 2009 | USA  (New York) | New York City Chinese Health Survey (NYC-CHS) | Cross-sectional | 2002-03 | N/A | Asian (Chinese) | All | *Continuous* | Smoking; Alcohol use;  Physical inactivity |
| 105 | Park et al. 2014 | USA | NLAAS [4] | Cross-sectional | 2002-03 | N/A | Asian | All | *Categorical*  0-10 years (Ref.)  11-20 years  21+ years | Alcohol use |
| 106 | Patel et al. 2018 | USA  (California) | California Asian Indian Tobacco Survey (CAITS) | Cross-sectional | 2004 | N/A | Asian (Indian) | All / Stratified | *Continuous* | Smoking |
| 107 | Patterson et al. 2013 | Canada | Canadian Community Health Survey - Mental Health (CCHS-MH) | Cross-sectional | 2002 | N/A | All (*unspecified*) | All | *Continuous* | Drug use |
| 108 | Perera et al. 2020 | USA | HCHS/SOL [22] | Cross-sectional | 2008-11 | N/A | Latino/Hispanic | All | *Categorical*  <10 years  10+ years  Native-born (Ref.) | Smoking;  Physical inactivity;  Diet |
| 109 | Reeske et al. 2009 | Germany | German Microcensus | Cross-sectional | 2005 | N/A | Middle Eastern (Turkish) | Stratified | *Categorical*  0-15 years  16-31 years  32+ years | Smoking |
| 110 | Reiss et al. 2014 | Netherlands  Germany | Permanent Research on Life Situations (POLS-Basic, Netherlands);  Microcensus (Germany) | Pooled cross-sectional | POLS-Basic: 1997-2004; Microcensus: 2009 | N/A | Middle Eastern (Turkish) | Stratified | *Categorical*  POLS-Basic:  <25 years  25+ years (Ref.);  Microcensus:  <10 years  10-29 years  30+ years (Ref.) | Smoking |
| 111 | Reiss et al. 2015 | Germany | Socio-Economic Panel Study (SOEP) | Pooled cross-sectional | 1998-2012 | N/A | European (Eastern);  Middle Eastern (Turkish) | Stratified | *Continuous* | Smoking |
| 112 | Riosmena et al. 2013 | USA | NHIS [14] (USA); Mexican Health and Aging Study (MHAS; Mexico) | Cross-sectional | 1997-2007 | N/A | Latino/Hispanic (Mexican) | Men | *Categorical*  <5 years (Ref.)  5-9 years  10-14 years  15+ years | Smoking |
| 113 | Riosmena et al. 2017 | USA | NHIS [14]; World Health Surveys (WHS) | Cross-sectional | NHIS: 2003-10; WHS: 2003-04 | N/A | All (*unspecified*) | Stratified | *Categorical*  <5 years  5-9 years  Native-born (Ref.) | Smoking |
| 114 | Salama et al. 2018 | Finland | Finnish Migrant Health and Wellbeing Study; Health 2011 Survey | Cross-sectional | 2010-12 | N/A | African (Somali);  European (Russian);  Middle Eastern (Kurdish) | Stratified | *Categorical*  <5 years  5+ years (Ref.) | Smoking;  Alcohol use;  Drug use |
| 115 | Salas-Wright et al. 2018 | USA | NESARC [12] | Cross-sectional | Wave 3: 2012-2013 | N/A | All (*unspecified*) | All | *Categorical*  <10 years  20+ years  Native-born (Ref.) | Substance use (alcohol/drug) |
| 116 | Sewali et al. 2015 | USA  (Minnesota) | New Americans Community Services (NACS) Survey | Cross-sectional | 2006-07 | N/A | African | All | *Categorical*  <5 years  5+ years (Ref.) | Smoking;  Physical inactivity |
| 117 | Singh & Siahpush 2002 | USA | NHIS [14] | Cross-sectional | 1993-94 | N/A | All (*unspecified*) | All | *Categorical*  <1 year  1-5 years  5-10 years  10-15 years  15+ years  Native-born (Ref.) | Smoking |
| 118 | So & Wong 2006 | USA | *Unnamed study (survey)* | Cross-sectional | *Not reported* | N/A | Asian/Pacific Islander | All | *Continuous* | Alcohol use;  Drug use |
| 119 | Sofianou et al. 2011 | USA | NHANES [8] | Pooled cross-sectional | 2003-04; 2005-06 | N/A | Latino/Hispanic (Mexican) | All | *Categorical*  <15 years  15+ years | Diet |
| 120 | Sordo et al. 2015 | Spain | Household Survey on Alcohol and Drugs in Spain (EDADES) | Cross-sectional | 2005-07 | N/A | European;  Latino/Hispanic;  Middle Eastern | All | *Categorical*  <5 years  5-9 years  10+ years  Native-born (Ref.) | Smoking;  Alcohol use;  Drug use |
| 121 | Takeuchi et al. 2007 | USA | NLAAS [4] | Cross-sectional | 2002-03 | N/A | Asian | Stratified | *Categorical*  <6 years  6-10 years  11-20 years  21+ years  Native-born (Ref.) | Substance use |
| 122 | Talegawkar et al. 2016 | USA  (California) | Mediators of Atherosclerosis in South Asians Living in America (MASALA) Study | Cross-sectional | Baseline:  2010-13 | N/A | Asian (Southern) | All | *Categorical*  Short (mean 15.1 years)  Medium (mean 27.1 years)  Long (mean 39.4 years) | Smoking;  Alcohol use; Physical inactivity;  Diet |
| 123 | Taylor et al. 2007 | USA  (Washington) | *Unnamed study (survey)* | Cross-sectional | 2005 | N/A | Asian (Chinese) | Men | *Categorical*  <10 years  10+ years | Smoking;  Physical inactivity |
| 124 | Terasaki et al. 2017 | USA | NIS [73] | Cross-sectional | 2003-04 | N/A | All (*unspecified*) | All | *Categorical*  <1 year (Ref.)  1-4 years  5-9 years  10-14 years  15+ years | Physical inactivity |
| 125 | Tortajada et al. 2010 | Spain | *Unnamed study (survey)* | Cross-sectional | 2006 | N/A | Latino/Hispanic (Latin American) | All | *Categorical*  <2 years  2+ years | Alcohol use |
| 126 | Tran et al. 2015 | Australia  (New South Wales) | 45 and Up Study | Cross-sectional | Baseline: 2006-08 | N/A | Asian (Vietnamese) | All | *Categorical*  <20 years (Ref.)  20-24 years  25+ years | Smoking;  Physical inactivity |
| 127 | Tremblay et al. 2006 | Canada | CCHS [34] | Pooled cross-sectional | 2000/01; 2003 | N/A | All (*unspecified*) | All / Stratified | *Categorical*  <10 years  10+ years  Native-born (Ref.) | Physical inactivity |
| 128 | Tseng & Fang 2011 | USA  (Pennsylvania) | *Unnamed study (survey)* | Cross-sectional | 2005-08 | N/A | Asian (Chinese) | Women | *Continuous* | Diet |
| 129 | Tseng et al. 2015 | USA  (Pennsylvania) | *Unnamed study (survey; same as [114])* | Cohort | Wave 1:  2005-08 Wave 2:  Until 2010 | 2 years | Asian (Chinese) | Women | *Continuous* | Diet |
| 130 | Van Hook et al. 2018 | USA | NHANES [8] | Pooled cross-sectional | 1999/2000- 2011/12 | N/A | Latino/Hispanic (Mexican) | All | *Continuous* | Diet |
| 131 | Vargas-Bustamante 2013 | USA | *Unnamed study (survey)* | Cross-sectional | 2010 | N/A | All (*unspecified*) | All | *Categorical*  <2 years  2-4 years  5-9 years  10-14 years  15+ years  Native-born (Ref.) | Physical inactivity |
| 132 | Vega & Sribney 2003 | USA | Mexican American Prevalence and Services Survey (MAPSS) | Cross-sectional | 1996 | N/A | Latino/Hispanic (Mexican) | Stratified | *Categorical*  <13 years  13+ years  Native-born (Ref.) | Alcohol use |
| 133 | Wong & Wong 2003 | Canada | Canadian National Population Health Survey (NPHS) | Cross-sectional | 1996-97 | N/A | All (*unspecified*) | Women | *Categorical*  0-4 years  5-9 years  10+ years | Smoking;  Physical inactivity |
| 134 | Wong et al. 2011 | USA | Southeast Asian Health Education Project (SEAHEP) | Cross-sectional | 2004-06 | N/A | Asian (Southeastern) | Women | *Categorical*  <20 years  20+ years | Smoking;  Alcohol use |
| 135 | Yang 2019 | Canada | CCHS-MH [93] | Cross-sectional | 2012 | N/A | All (*unspecified*) | All | *Continuous* | Smoking;  Drug use; Physical inactivity |
| 136 | Yang et al. 2007 | USA | *Unnamed study (survey)* | Cross-sectional | 2000 | N/A | Asian (Korean) | Stratified | *Categorical*  0-15 years  16-25 years  26+ years | Alcohol use |
| 137 | Zan & Fan 2018 | USA | NHANES [8] | Cross-sectional | 2003-06 | N/A | All (*unspecified*) | Stratified | *Categorical*  <10 years  10+ years  Native-born (Ref.) | Physical inactivity |

**Table 3.** Quality assessment: Cross-sectional studies

| **Ref.** | **Authors, Year** | **Selection** | | | | | | | | | | |
| --- | --- | --- | --- | --- | --- | --- | --- | --- | --- | --- | --- | --- |
|  |  | **Representativeness** | | | | **Sample Size** | | **Non-respondents** | | | **Ascertainment of exposure** | |
|  |  | Truly (1 star) | Somewhat (1 star) | Selected (0 stars) | No description (0 stars) | Justified (1 star) | Not justified  (0 stars) | Respondent/ non-respondent comparability/ weighting and response rate satisfactory  (1 star) | Respondent/ non-respondent comparability or response rate unsatisfactory  (0 stars) | No description of respondent/ non-respondent characteristics or response rate  (0 stars) | Validated measurement tool or justified categories  (1 star) | Non-validated measurement tool, non-justified categories, or no description of measurement tool  (0 stars) |
| 15 | Addo et al. 2018 |  | 1 |  |  | 1 |  |  |  | 0 | 1 |  |
| 16 | Afable et al. 2016 |  | 1 |  |  |  | 0 |  |  | 0 | 1 |  |
| 17 | Agic et al. 2016 | 1 |  |  |  | 1 |  | 1 |  |  | 1 |  |
| 18 | Alegria et al. 2007 | 1 |  |  |  | 1 |  | 1 |  |  | 1 |  |
| 19 | Amundsen 2012 | 1 |  |  |  | 1 |  | 1 |  |  |  | 0 |
| 20 | An et al. 2008 | 1 |  |  |  | 1 |  |  | 0 |  |  | 0 |
| 21 | Azagba et al. 2019 | 1 |  |  |  | 1 |  | 1 |  |  |  | 0 |
| 22 | Basu et al. 2019 | 1 |  |  |  | 1 |  | 1 |  |  |  | 0 |
| 23 | Bayog & Waters 2018 | 1 |  |  |  | 1 |  | 1 |  |  | 1 |  |
| 24 | Bharmal et al. 2015 | 1 |  |  |  | 1 |  | 1 |  |  | 1 |  |
| 25 | Bingham et al. 2016 |  | 1 |  |  |  | 0 |  |  | 0 |  | 0 |
| 26 | Blanco et al. 2013 | 1 |  |  |  | 1 |  |  |  | 0 |  | 0 |
| 27 | Borges et al. 2006 | 1 |  |  |  | 1 |  | 1 |  |  | 1 |  |
| 29 | Brathwaite et al. 2017 |  | 1 |  |  | 1 |  |  |  | 0 | 1 |  |
| 30 | Breslau et al. 2006 | 1 |  |  |  | 1 |  | 1 |  |  | 1 |  |
| 31 | Breslau et al. 2007 | 1 |  |  |  | 1 |  | 1 |  |  | 1 |  |
| 32 | Brock et al. 2001 |  |  | 0 |  |  | 0 |  |  | 0 |  | 0 |
| 33 | Brown et al. 2018 | 1 |  |  |  | 1 |  |  |  | 0 | 1 |  |
| 34 | Canfield et al. 2017 |  |  | 0 |  |  | 0 |  |  | 0 |  | 0 |
| 35 | Cano et al. 2017 |  |  | 0 |  | 1 |  |  |  | 0 |  | 0 |
| 36 | Castañeda et al. 2019 |  | 1 |  |  | 1 |  | 1 |  |  |  | 0 |
| 38 | Choi et al. 2008 |  | 1 |  |  |  | 0 |  |  | 0 |  | 0 |
| 39 | Chou et al. 2010 |  | 1 |  |  | 1 |  |  |  | 0 | 1 |  |
| 40 | Commodore-Mensah et al. 2018 |  |  | 0 |  |  | 0 |  |  | 0 | 1 |  |
| 41 | Constantine et al. 2013 |  | 1 |  |  | 1 |  | 1 |  |  |  | 0 |
| 42 | Coronado et al. 2008 |  | 1 |  |  | 1 |  | 1 |  |  | 1 |  |
| 43 | Creighton et al. 2012 | 1 |  |  |  | 1 |  | 1 |  |  |  | 0 |
| 44 | Crespo et al. 2001 | 1 |  |  |  | 1 |  | 1 |  |  | 1 |  |
| 45 | Dawson et al. 2005 | 1 |  |  |  | 1 |  |  | 0 |  | 1 |  |
| 46 | de Munter et al. 2013 | 1 |  |  |  | 1 |  | 1 |  |  | 1 |  |
| 47 | Delisle et al. 2009 |  | 1 |  |  |  | 0 | 1 |  |  | 1 |  |
| 48 | Dogra et al. 2010 | 1 |  |  |  | 1 |  | 1 |  |  |  | 0 |
| 49 | Echeverria et al. 2013 | 1 |  |  |  | 1 |  |  | 0 |  |  | 0 |
| 50 | Ertl et al. 2018 |  | 1 |  |  | 1 |  |  |  | 0 |  | 0 |
| 51 | Evenson 2004 |  |  | 0 |  |  | 0 |  |  | 0 |  | 0 |
| 52 | Gaskins et al. 2012 | 1 |  |  |  | 1 |  | 1 |  |  |  | 0 |
| 53 | Gazard et al. 2015 | 1 |  |  |  | 1 |  | 1 |  |  | 1 |  |
| 54 | Glenn et al. 2009 |  | 1 |  |  |  | 0 |  | 0 |  |  | 0 |
| 55 | Goff et al. 2015 |  | 1 |  |  |  | 0 |  |  | 0 | 1 |  |
| 56 | Gorman et al. 2014 | 1 |  |  |  | 1 |  | 1 |  |  | 1 |  |
| 57 | Ham et al. 2007 | 1 |  |  |  | 1 |  | 1 |  |  | 1 |  |
| 58 | Hardie et al. 2013 |  |  | 0 |  |  | 0 |  |  | 0 |  | 0 |
| 59 | Hislop et al. 2008 | 1 |  |  |  | 1 |  |  | 0 |  |  | 0 |
| 60 | Holmes & Marcelli 2014 | 1 |  |  |  |  | 0 |  |  | 0 |  | 0 |
| 61 | Hu et al. 2010 | 1 |  |  |  |  | 0 |  |  | 0 | 1 |  |
| 62 | Huffman et al. 2014 |  | 1 |  |  | 1 |  |  |  | 0 |  | 0 |
| 63 | Hyman et al. 2008 |  | 1 |  |  | 1 |  |  | 0 |  |  | 0 |
| 64 | Jayaweera & Quigley 2010 | 1 |  |  |  | 1 |  | 1 |  |  | 1 |  |
| 65 | Jin et al. 2017 | 1 |  |  |  | 1 |  |  |  | 0 | 1 |  |
| 66 | Johnson et al. 2002 | 1 |  |  |  | 1 |  | 1 |  |  | 1 |  |
| 67 | Jonnalagadda & Diwan 2005 |  | 1 |  |  |  | 0 |  | 0 |  |  | 0 |
| 70 | Juon et al. 2003 |  | 1 |  |  |  | 0 |  | 0 |  | 1 |  |
| 71 | Kabir et al. 2008 |  |  | 0 |  | 1 |  |  |  | 0 | 1 |  |
| 72 | Kandula & Lauderdale 2005 | 1 |  |  |  | 1 |  | 1 |  |  | 1 |  |
| 73 | Kershaw et al. 2016 |  | 1 |  |  | 1 |  |  | 0 |  |  | 0 |
| 75 | Kim et al. 2014 |  |  | 0 |  | 1 |  |  |  | 0 |  | 0 |
| 76 | Koca & Lapa 2014 |  |  | 0 |  | 1 |  |  |  | 0 |  | 0 |
| 77 | Kolar et al. 2018 |  |  | 0 |  | 1 |  |  |  | 0 |  | 0 |
| 78 | Koya & Egede 2007 | 1 |  |  |  | 1 |  | 1 |  |  | 1 |  |
| 79 | Kuerban 2016 | 1 |  |  |  | 1 |  |  |  | 0 | 1 |  |
| 80 | Lacey et al. 2015 | 1 |  |  |  | 1 |  | 1 |  |  |  | 0 |
| 81 | Lacey et al. 2016 | 1 |  |  |  | 1 |  | 1 |  |  | 1 |  |
| 82 | Lear et al. 2009 |  |  | 0 |  |  | 0 |  |  | 0 | 1 |  |
| 83 | Lee et al. 2007 |  |  | 0 |  |  | 0 |  |  | 0 |  | 0 |
| 84 | Leshem & Dessie-Navon 2018 |  |  |  | 0 | 1 |  |  |  | 0 |  | 0 |
| 85 | Leung 2014 | 1 |  |  |  | 1 |  |  |  | 0 |  | 0 |
| 86 | Levy et al. 2005 |  |  | 0 |  |  | 0 | 1 |  |  |  | 0 |
| 87 | Li & Hummer 2015 | 1 |  |  |  | 1 |  | 1 |  |  |  | 0 |
| 88 | Li & Wen 2013 | 1 |  |  |  | 1 |  | 1 |  |  |  | 0 |
| 89 | Li & Wen 2015 | 1 |  |  |  | 1 |  | 1 |  |  | 1 |  |
| 90 | Lo et al. 2014 | 1 |  |  |  | 1 |  |  |  | 0 |  | 0 |
| 91 | Lopez-Gonzalez et al. 2005 |  | 1 |  |  | 1 |  |  |  | 0 |  | 0 |
| 92 | Maffini et al. 2015 | 1 |  |  |  | 1 |  | 1 |  |  |  | 0 |
| 93 | Mahmood et al. 2019 | 1 |  |  |  | 1 |  |  | 0 |  | 1 |  |
| 94 | Marin-Guerrero et al. 2015 | 1 |  |  |  | 1 |  |  |  | 0 | 1 |  |
| 95 | Maxwell et al. 2005 | 1 |  |  |  | 1 |  |  | 0 |  |  | 0 |
| 96 | Maxwell et al. 2007 |  | 1 |  |  |  | 0 |  |  | 0 |  | 0 |
| 97 | Mejean et al. 2009 |  | 1 |  |  |  | 0 |  |  | 0 | 1 |  |
| 98 | Meshefedjian et al. 2014 | 1 |  |  |  | 1 |  | 1 |  |  |  | 0 |
| 99 | Murillo et al. 2015 | 1 |  |  |  | 1 |  |  |  | 0 |  | 0 |
| 100 | Nicolaou et al. 2006 |  | 1 |  |  |  | 0 | 1 |  |  | 1 |  |
| 101 | Osei-Kwasi et al. 2019 | 1 |  |  |  | 1 |  |  |  | 0 | 1 |  |
| 102 | Osypuk et al. 2009 |  | 1 |  |  | 1 |  |  |  | 0 | 1 |  |
| 103 | Pampel et al. 2019 | 1 |  |  |  | 1 |  | 1 |  |  | 1 |  |
| 104 | Parikh et al. 2009 | 1 |  |  |  | 1 |  | 1 |  |  |  | 0 |
| 105 | Park et al. 2014 | 1 |  |  |  | 1 |  | 1 |  |  | 1 |  |
| 106 | Patel et al. 2018 |  | 1 |  |  | 1 |  |  | 0 |  |  | 0 |
| 107 | Patterson et al. 2013 |  | 1 |  |  | 1 |  | 1 |  |  |  | 0 |
| 108 | Perera et al. 2019 |  | 1 |  |  | 1 |  | 1 |  |  |  | 0 |
| 109 | Reeske et al. 2009 | 1 |  |  |  | 1 |  | 1 |  |  | 1 |  |
| 110 | Reiss et al. 2014 | 1 |  |  |  | 1 |  | 1 |  |  |  | 0 |
| 111 | Reiss et al. 2015 | 1 |  |  |  | 1 |  | 1 |  |  | 1 |  |
| 112 | Riosmena et al. 2013 | 1 |  |  |  | 1 |  |  | 0 |  | 1 |  |
| 113 | Riosmena et al. 2017 | 1 |  |  |  |  | 0 |  | 0 |  | 1 |  |
| 114 | Salama et al. 2018 | 1 |  |  |  | 1 |  | 1 |  |  |  | 0 |
| 115 | Salas-Wright et al. 2018 | 1 |  |  |  | 1 |  | 1 |  |  |  | 0 |
| 116 | Sewali et al. 2015 |  | 1 |  |  | 1 |  |  | 0 |  |  | 0 |
| 117 | Singh & Siahpush 2002 | 1 |  |  |  | 1 |  |  |  | 0 | 1 |  |
| 118 | So & Wong 2006 |  |  | 0 |  |  | 0 |  |  | 0 |  | 0 |
| 119 | Sofianou et al. 2011 | 1 |  |  |  | 1 |  | 1 |  |  |  | 0 |
| 120 | Sordo et al. 2015 | 1 |  |  |  | 1 |  |  | 0 |  | 1 |  |
| 121 | Takeuchi et al. 2007 | 1 |  |  |  |  | 0 | 1 |  |  | 1 |  |
| 122 | Talegawkar et al. 2016 |  |  | 0 |  |  | 0 | 1 |  |  |  | 0 |
| 123 | Taylor et al. 2007 |  | 1 |  |  | 1 |  |  | 0 |  |  | 0 |
| 124 | Terasaki et al. 2017 | 1 |  |  |  | 1 |  |  | 0 |  | 1 |  |
| 125 | Tortajada et al. 2010 |  | 1 |  |  | 1 |  |  |  | 0 |  | 0 |
| 126 | Tran et al. 2015 |  | 1 |  |  | 1 |  |  | 0 |  | 1 |  |
| 127 | Tremblay et al. 2006 | 1 |  |  |  | 1 |  |  | 0 |  |  | 0 |
| 128 | Tseng & Fang 2011 |  | 1 |  |  |  | 0 |  |  | 0 |  | 0 |
| 130 | Van Hook et al. 2018 | 1 |  |  |  | 1 |  |  |  | 0 |  | 0 |
| 131 | Vargas-Bustamante 2013 |  |  | 0 |  |  | 0 |  |  | 0 | 1 |  |
| 132 | Vega & Sribney 2003 | 1 |  |  |  | 1 |  |  | 0 |  | 1 |  |
| 133 | Wong & Wong 2003 | 1 |  |  |  | 1 |  |  |  | 0 | 1 |  |
| 134 | Wong et al. 2011 |  |  | 0 |  |  | 0 |  |  | 0 | 1 |  |
| 135 | Yang 2019 | 1 |  |  |  | 1 |  | 1 |  |  |  | 0 |
| 136 | Yang et al. 2007 |  | 1 |  |  | 1 |  |  | 0 |  | 1 |  |
| 137 | Zan & Fan 2018 | 1 |  |  |  | 1 |  |  |  | 0 | 1 |  |

**Table 4.** Quality assessment: Cross-sectional studies (*continued*)

| **Ref.** | **Authors, Year** | **Comparability** | | **Outcome** | | | | | **Final score** | **Percentage score** |
| --- | --- | --- | --- | --- | --- | --- | --- | --- | --- | --- |
|  |  | **Comparability of groups** | | **Assessment of outcome** | | | **Statistical test** | |  |  |
|  |  | Controls for or matches on age/sex (1 star) | Controls for or matches on other factors (e.g., SES) (1 star) | Most reliable assessment method  (2 stars) | Semi-reliable assessment method (1 star) | No description (0 stars) | Statistical test clearly described and appropriate, association presented with confidence interval and/or p-value (1 star) | Statistical test not appropriate, not described or incomplete (0 stars) | Summed score | Divide summed score by 9 |
| 15 | Addo et al. 2018 | 1 | 1 | 2 |  |  | 1 |  | 8 | 89% |
| 16 | Afable et al. 2016 | 1 | 1 | 2 |  |  | 1 |  | 7 | 78% |
| 17 | Agic et al. 2016 | 0 | 0 | 2 |  |  | 1 |  | 7 | 78% |
| 18 | Alegria et al. 2007a | 1 | 0 | 2 |  |  | 1 |  | 8 | 89% |
| 19 | Amundsen 2012 | 1 | 1 | 2 |  |  | 1 |  | 8 | 89% |
| 20 | An et al. 2008 | 1 | 1 | 2 |  |  | 1 |  | 8 | 89% |
| 21 | Azagba et al. 2019 | 1 | 1 |  | 1 |  | 1 |  | 7 | 78% |
| 22 | Basu et al. 2019 | 1 | 1 |  | 1 |  | 1 |  | 7 | 78% |
| 23 | Bayog & Waters 2018 | 1 | 0 |  | 1 |  | 1 |  | 7 | 78% |
| 24 | Bharmal et al. 2015 | 1 | 1 |  | 1 |  | 1 |  | 8 | 89% |
| 25 | Bingham et al. 2016 | 0 | 0 | 2 |  |  |  | 0 | 3 | 33% |
| 26 | Blanco et al. 2013 | 1 | 1 | 2 |  |  | 1 |  | 7 | 78% |
| 27 | Borges et al. 2006 | 1 | 1 | 2 |  |  | 1 |  | 9 | 100% |
| 29 | Brathwaite et al. 2017 | 1 | 1 |  | 1 |  | 1 |  | 7 | 78% |
| 30 | Breslau et al. 2006 | 1 | 0 | 2 |  |  | 1 |  | 8 | 89% |
| 31 | Breslau et al. 2007 | 1 | 00 | 2 |  |  | 1 |  | 8 | 89% |
| 32 | Brock et al. 2001 | 1 | 1 |  |  | 0 | 1 |  | 3 | 33% |
| 33 | Brown et al. 2018 | 1 | 1 | 2 |  |  | 1 |  | 8 | 89% |
| 34 | Canfield et al. 2017 | 1 | 1 | 2 |  |  |  | 0 | 4 | 44% |
| 35 | Cano et al. 2017 | 1 | 1 | 2 |  |  | 1 |  | 6 | 67% |
| 36 | Castañeda et al. 2019 | 1 | 1 | 2 |  |  | 1 |  | 8 | 89% |
| 38 | Choi et al. 2008 | 0 | 0 | 2 |  |  | 1 |  | 4 | 44% |
| 39 | Chou et al. 2010 | 1 | 1 |  | 1 |  | 1 |  | 7 | 78% |
| 40 | Commodore-Mensah et al. 2018 | 1 | 1 | 2 |  |  | 1 |  | 6 | 67% |
| 41 | Constantine et al. 2013 | 0 | 0 | 2 |  |  | 1 |  | 6 | 67% |
| 42 | Coronado et al. 2008 | 1 | 1 | 2 |  |  | 1 |  | 9 | 100% |
| 43 | Creighton et al. 2012 | 1 | 1 | 2 |  |  | 1 |  | 8 | 89% |
| 44 | Crespo et al. 2001 | 1 | 1 | 2 |  |  | 1 |  | 9 | 100% |
| 45 | Dawson et al. 2005 | 1 | 1 | 2 |  |  | 1 |  | 8 | 89% |
| 46 | de Munter et al. 2013 | 0 | 1 | 2 |  |  | 1 |  | 8 | 89% |
| 47 | Delisle et al. 2009 | 1 | 0 | 2 |  |  | 1 |  | 6 | 67% |
| 48 | Dogra et al. 2010 | 1 | 0 |  | 1 |  | 1 |  | 6 | 67% |
| 49 | Echeverria et al. 2013 | 1 | 1 |  | 1 |  | 1 |  | 6 | 67% |
| 50 | Ertl et al. 2018 | 0 | 0 | 2 |  |  | 1 |  | 5 | 56% |
| 51 | Evenson 2004 | 1 | 1 | 1 |  |  | 1 |  | 4 | 44% |
| 52 | Gaskins et al. 2012 | 1 | 1 | 2 |  |  | 1 |  | 8 | 89% |
| 53 | Gazard et al. 2015 | 1 | 1 | 2 |  |  | 1 |  | 9 | 100% |
| 54 | Glenn et al. 2009 | 1 | 1 | 2 |  |  | 1 |  | 6 | 67% |
| 55 | Goff et al. 2015 | 1 | 0 | 2 |  |  |  | 0 | 5 | 56% |
| 56 | Gorman et al. 2014 | 1 | 1 | 2 |  |  | 1 |  | 9 | 100% |
| 57 | Ham et al. 2007 | 1 | 1 |  | 1 |  | 1 |  | 8 | 89% |
| 58 | Hardie et al. 2013 | 1 | 0 | 2 |  |  | 1 |  | 4 | 44% |
| 59 | Hislop et al. 2008 | 1 | 1 | 2 |  |  | 1 |  | 7 | 78% |
| 60 | Holmes & Marcelli 2014 | 1 | 1 |  | 1 |  | 1 |  | 5 | 56% |
| 61 | Hu et al. 2010 | 1 | 1 |  | 1 |  | 1 |  | 6 | 67% |
| 62 | Huffman et al. 2014 | 1 | 1 | 2 |  |  | 1 |  | 7 | 78% |
| 63 | Hyman et al. 2008 | 0 | 0 |  | 1 |  | 1 |  | 4 | 44% |
| 64 | Jayaweera & Quigley 2010 | 1 | 1 |  | 1 |  | 1 |  | 8 | 89% |
| 65 | Jin et al. 2017 | 1 | 1 |  | 1 |  | 1 |  | 7 | 78% |
| 66 | Johnson et al. 2002 | 1 | 1 |  | 1 |  | 1 |  | 8 | 89% |
| 67 | Jonnalagadda & Diwan 2005 | 1 | 1 |  | 1 |  | 1 |  | 5 | 56% |
| 70 | Juon et al. 2003 | 1 | 1 |  | 1 |  | 1 |  | 6 | 67% |
| 71 | Kabir et al. 2008 | 1 | 1 |  | 1 |  | 1 |  | 6 | 67% |
| 72 | Kandula & Lauderdale 2005 | 1 | 0 | 2 |  |  | 1 |  | 8 | 89% |
| 73 | Kershaw et al. 2016 | 0 | 0 | 2 |  |  |  | 0 | 4 | 44% |
| 75 | Kim et al. 2014 | 0 | 0 | 2 |  |  | 1 |  | 4 | 44% |
| 76 | Koca & Lapa 2014 | 1 | 1 | 2 |  |  | 1 |  | 6 | 67% |
| 77 | Kolar et al. 2018 | 1 | 0 | 2 |  |  | 1 |  | 5 | 56% |
| 78 | Koya & Egede 2007 | 1 | 1 | 2 |  |  | 1 |  | 9 | 100% |
| 79 | Kuerban 2016 | 1 | 1 |  | 1 |  | 1 |  | 7 | 78% |
| 80 | Lacey et al. 2015 | 1 | 1 | 2 |  |  | 1 |  | 8 | 89% |
| 81 | Lacey et al. 2016 | 0 | 0 | 2 |  |  | 1 |  | 7 | 78% |
| 82 | Lear et al. 2009 | 0 | 0 |  | 1 |  |  | 0 | 2 | 22% |
| 83 | Lee et al. 2007 | 1 | 0 |  | 1 |  | 1 |  | 3 | 33% |
| 84 | Leshem & Dessie-Navon 2018 | 1 | 0 | 2 |  |  |  | 0 | 4 | 44% |
| 85 | Leung 2014 | 1 | 1 |  | 1 |  | 1 |  | 6 | 67% |
| 86 | Levy et al. 2005 | 0 | 0 |  |  | 0 |  | 0 | 1 | 11% |
| 87 | Li & Hummer 2015 | 1 | 1 |  | 1 |  | 1 |  | 7 | 78% |
| 88 | Li & Wen 2013 | 1 | 1 |  | 1 |  | 1 |  | 7 | 78% |
| 89 | Li & Wen 2015 | 1 | 1 |  | 1 |  | 1 |  | 8 | 89% |
| 90 | Lo et al. 2014 | 1 | 1 |  | 1 |  | 1 |  | 6 | 67% |
| 91 | Lopez-Gonzalez et al. 2005 | 1 | 1 |  | 1 |  | 1 |  | 6 | 67% |
| 92 | Maffini et al. 2015 | 1 | 1 |  | 1 |  | 1 |  | 7 | 78% |
| 93 | Mahmood et al. 2019 | 1 | 1 | 2 |  |  | 1 |  | 8 | 89% |
| 94 | Marin-Guerrero et al. 2015 | 1 | 1 | 2 |  |  | 1 |  | 8 | 89% |
| 95 | Maxwell et al. 2005 | 0 | 0 |  | 1 |  |  | 0 | 3 | 33% |
| 96 | Maxwell et al. 2007 | 1 | 1 |  | 1 |  | 1 |  | 5 | 56% |
| 97 | Mejean et al. 2009 | 1 | 1 | 2 |  |  | 1 |  | 7 | 78% |
| 98 | Meshefedjian et al. 2014 | 1 | 1 |  | 1 |  | 1 |  | 7 | 78% |
| 99 | Murillo et al. 2015 | 1 | 1 | 2 |  |  | 1 |  | 7 | 78% |
| 100 | Nicolaou et al. 2006 | 1 | 0 |  | 1 |  | 1 |  | 6 | 67% |
| 101 | Osei-Kwasi et al. 2019 | 1 | 1 | 2 |  |  | 1 |  | 8 | 89% |
| 102 | Osypuk et al. 2009 | 1 | 1 | 2 |  |  | 1 |  | 8 | 89% |
| 103 | Pampel et al. 2019 | 1 | 1 |  | 1 |  | 1 |  | 8 | 89% |
| 104 | Parikh et al. 2009 | 1 | 1 | 2 |  |  | 1 |  | 8 | 89% |
| 105 | Park et al. 2014 | 1 | 1 | 2 |  |  | 1 |  | 9 | 100% |
| 106 | Patel et al. 2018 | 1 | 1 |  | 1 |  | 1 |  | 6 | 67% |
| 107 | Patterson et al. 2013 | 1 | 1 |  | 1 |  | 1 |  | 7 | 78% |
| 108 | Perera et al. 2019 | 0 | 0 |  | 1 |  | 1 |  | 5 | 56% |
| 109 | Reeske et al. 2009 | 1 | 1 |  | 1 |  | 1 |  | 8 | 89% |
| 110 | Reiss et al. 2014 | 1 | 1 |  | 1 |  | 1 |  | 7 | 78% |
| 111 | Reiss et al. 2015 | 1 | 1 |  | 1 |  |  | 0 | 7 | 78% |
| 112 | Riosmena et al. 2017 | 1 | 1 |  | 1 |  | 1 |  | 6 | 67% |
| 113 | Riosmena et al. 2013 | 1 | 1 |  |  | 0 |  | 0 | 5 | 56% |
| 114 | Salama et al. 2018 | 1 | 1 | 2 |  |  | 1 |  | 8 | 89% |
| 115 | Salas-Wright et al. 2018 | 1 | 1 |  | 1 |  | 1 |  | 7 | 78% |
| 116 | Sewali et al. 2015 | 1 | 1 |  | 1 |  | 1 |  | 6 | 67% |
| 117 | Singh & Siahpush 2002 | 1 | 1 |  |  | 0 | 1 |  | 6 | 67% |
| 118 | So & Wong 2006 | 0 | 0 |  | 1 |  |  | 0 | 1 | 11% |
| 119 | Sofianou et al. 2011 | 1 | 1 | 2 |  |  |  | 0 | 7 | 78% |
| 120 | Sordo et al. 2015 | 1 | 1 |  | 1 |  | 1 |  | 7 | 78% |
| 121 | Takeuchi et al. 2007 | 1 | 0 | 2 |  |  | 1 |  | 7 | 78% |
| 122 | Talegawkar et al. 2016 | 0 | 0 | 2 |  |  |  | 0 | 3 | 33% |
| 123 | Taylor et al. 2007 | 1 | 1 |  | 1 |  |  | 0 | 5 | 56% |
| 124 | Terasaki et al. 2017 | 1 | 1 |  | 1 |  | 1 |  | 7 | 78% |
| 125 | Tortajada et al. 2010 | 0 | 0 | 2 |  |  |  | 0 | 4 | 44% |
| 126 | Tran et al. 2015 | 1 | 1 |  | 1 |  | 1 |  | 7 | 78% |
| 127 | Tremblay et al. 2006 | 0 | 0 |  | 1 |  |  | 0 | 3 | 33% |
| 128 | Tseng & Fang 2011 | 0 | 0 | 2 |  |  | 1 |  | 4 | 44% |
| 130 | Van Hook et al. 2018 | 1 | 1 | 2 |  |  | 1 |  | 7 | 78% |
| 131 | Vargas-Bustamante 2013 | 1 | 0 |  | 1 |  | 1 |  | 4 | 44% |
| 132 | Vega & Sribney 2003 | 1 | 0 | 2 |  |  |  | 0 | 5 | 56% |
| 133 | Wong & Wong 2003 | 0 | 0 |  |  | 0 | 1 |  | 4 | 44% |
| 134 | Wong et al. 2011 | 1 | 1 |  |  | 0 |  | 0 | 3 | 33% |
| 135 | Yang 2019 | 1 | 1 |  | 1 |  | 1 |  | 7 | 78% |
| 136 | Yang et al. 2007 | 1 | 0 | 2 |  |  | 1 |  | 7 | 78% |
| 137 | Zan & Fan 2018 | 1 | 1 | 2 |  |  | 1 |  | 8 | 89% |

**Table 4.** Quality assessment: Cohort studies

| **Ref.** | **Authors, Year** | **Selection** | | | | | | | | | | |
| --- | --- | --- | --- | --- | --- | --- | --- | --- | --- | --- | --- | --- |
|  |  | **Representativeness** | | | | **Non-exposed cohort** | | | **Ascertainment of exposure** | | **Outcome at baseline** | |
|  |  | Truly  (1 star) | Somewhat  (1 star) | Selected  (0 stars) | No description  (0 stars) | Same as exposed (1 star) | Different than exposed (0 stars) | No description  (0 stars) | Non-validated, but available and described  (1 star) | No description  (0 stars) | Demonstration that outcome not present at study baseline (1 star) | No demonstration (0 stars) |
| 28 | Bostean et al. 2017 | 1 |  |  |  | 1 |  |  | 1 |  |  | 0 |
| 37 | Chartier et al. 2017 | 1 |  |  |  | 1 |  |  | 1 |  |  | 0 |
| 68 | Joshi et al. 2017 | 1 |  |  |  | 1 |  |  | 1 |  | 1 |  |
| 69 | Joshi et al. 2018 | 1 |  |  |  | 1 |  |  | 1 |  | 1 |  |
| 74 | Khlat et al. 2019 | 1 |  |  |  | 1 |  |  |  | 0 | 1 |  |
| 129 | Tseng et al. 2015 |  | 1 |  |  |  |  | 0 |  | 0 | 1 |  |

**Table 5.** Quality assessment: Cohort studies (*continued*)

| **Ref.** | **Authors, Year** | **Comparability** | | **Outcome** | | | | | | | | | **Final score** | **Percentage score** |
| --- | --- | --- | --- | --- | --- | --- | --- | --- | --- | --- | --- | --- | --- | --- |
|  |  | **Comparability of groups** | | **Assessment of outcome** | | | **Follow-up** | | **Adequacy of follow-up** | | | |  |  |
|  |  | Controls for or matches on age and sex (1 star) | Controls for or matches on other factors (e.g., SES) (1 star) | Most reliable assessment method  (2 stars) | Semi-reliable assessment method (1 star) | No description (0 stars) | Long enough for outcomes to occur (1 star) | Not sufficiently long (0 stars) | Complete follow-up (1 star) | Subjects lost to follow up unlikely to introduce bias, or description provided of those lost (1 star) | Low follow-up rate and no description of those lost (0 stars) | No statement (0 stars) | Summed score | Divide summed score by 11 |
| 28 | Bostean et al. 2017 | 1 | 1 | 2 |  |  | 1 |  |  |  |  | 0 | 8 | 73% |
| 37 | Chartier et al. 2017 | 1 | 1 | 2 |  |  | 1 |  |  | 1 |  |  | 9 | 82% |
| 68 | Joshi et al., 2017 | 1 | 1 | 2 |  |  | 1 |  |  | 1 |  |  | 10 | 91% |
| 69 | Joshi et al., 2018 | 1 | 1 |  | 1 |  | 1 |  |  | 1 |  |  | 9 | 82% |
| 74 | Khlat et al. 2019 | 1 | 1 |  | 1 |  | 1 |  |  | 1 |  |  | 8 | 73% |
| 129 | Tseng et al. 2015 | 0 | 0 | 2 |  |  | 1 |  |  | 1 |  |  | 6 | 55% |

**Table 6.** Index of included studies

| ***By health risk behaviour*** | |
| --- | --- |
| Smoking | 20, 21, 23, 25, 28, 29, 32-34, 39, 41, 42, 49, 53, 54, 56, 59-61, 63-65, 67, 69-71, 74, 78, 79, 82, 84, 85, 87, 89, 91, 92, 95, 96, 98, 103, 104, 106, 108-114, 116, 117, 120, 122, 123, 126, 133-135 |
| Alcohol consumption | 15, 17, 19, 24, 25, 27, 32, 34-37, 50, 53, 58, 64, 66, 75, 89-91, 98, 101, 104, 105, 114, 118, 120, 122, 125, 134, 136 |
| Drug use | 34, 35, 53, 66, 77, 86, 114, 118, 120, 135 |
| Substance use diagnoses or dependence | 18, 26, 27, 30, 31, 80, 81, 107, 121 |
| Physical inactivity | 16, 22-25, 32, 33, 38, 40, 42-46, 48, 49, 51, 52, 57, 59, 65, 67, 68, 72, 73, 76, 78, 82-84, 87, 88, 93, 97-99, 102, 104, 108, 116, 122-124, 126, 127, 131, 133, 135, 137 |
| Diet | 22, 33, 47, 55, 62, 73, 82-84, 94, 100, 108, 119, 122, 128-130 |
| ***By study design*** | |
| Cross-sectional | 15-27, 29-36, 38-67, 70-73, 75-128, 130-137 |
| Longitudinal | 28, 37, 68, 69, 74, 129 |
| ***By study context*** | |
| **North America** |  |
| USA | 16, 18, 20-28, 30, 31, 33, 35-44, 49-52, 54, 56-58, 60-62, 66, 67, 70, 72, 73, 75, 78-81, 85-92, 95, 96, 99, 102-106, 108, 112, 113, 115-119, 121-124, 128-132, 134, 136, 137 |
| Canada | 17, 48, 59, 63, 77, 82, 93, 98, 107, 127, 133, 135 |
| **Europe** |  |
| Finland | 114 |
| France | 74, 97 |
| Germany | 15, 29, 76, 101, 109-111 |
| Ireland | 71 |
| The Netherlands | 15, 29, 46, 100, 101, 110 |
| Norway | 19 |
| Spain | 47, 94, 120, 125 |
| Sweden | 45 |
| UK | 15, 29, 34, 53, 55, 64, 101 |
| **Other** |  |
| Australia | 32, 65, 68, 69, 83, 126 |
| Israel | 84 |
| ***By immigrant origin*** | |
| Asian | 16, 20, 23, 24, 31, 32, 38, 39, 41, 42, 46, 54, 56, 59, 61, 65, 67, 70, 72, 75, 79, 82, 83, 87, 90, 92, 96, 100, 102, 104-106, 118, 121-123, 126, 128, 129, 134, 136 |
| African/Afro-Caribbean | 15, 25, 29, 33, 40, 46, 47, 55, 62, 63, 74, 80, 81, 84, 100, 101, 114, 116, 120 |
| Latino/Hispanic | 18, 26-28, 34-37, 41, 43, 44, 49-51, 56-58, 60, 73, 86, 94, 95, 99, 102, 108, 113, 119, 120, 125, 130, 132 |
| European | 71, 82, 111, 114, 120 |
| Middle Eastern | 19, 76, 97, 109-111, 114, 120 |
| ***By sex*** | |
| Stratified | 15, 18, 20, 21, 27, 28, 36, 40, 41, 45, 48, 56, 63, 65, 72-75, 78, 89, 91, 95, 100, 103, 106, 109-112, 114, 121, 127, 132, 136, 137 |
| Men only | 29, 32, 54, 59, 61, 70, 86, 96, 97, 104, 113, 123, 126 |
| Women only | 38, 42, 50-52, 58, 64, 83, 128, 129, 133, 134 |

**Tables 7-19.** Narrative synthesis of the 123 studies included in the systematic review

**Smoking (58 studies)**

**Table 7. All smoking studies**

| **Region** | **Sex** | **Increased risk** | **Non-significant increased risk** | **No difference**  **(i.e., OR=1.00)** | **Non-significant decreased risk** | **Decreased risk** | **No trend** |
| --- | --- | --- | --- | --- | --- | --- | --- |
| **North America**  **(USA, Canada)** | **All** | **All migrants (2)**  Koya & Egede 2007 [Adjusted]  Leung 2014  **African/Caribbean (1)**  Brown et al. 2018  **Asian (3)**  Li & Hummer 2015  Maffini et al. 2015  Patel et al. 2018 | **All migrants (5)**  Gorman et al. 2014  Lopez-Gonzalez et al. 2005  Meshefedjian et al. 2014 [Adjusted]  Singh & Siahpush 2002  Yang 2019  **African (1)**  Sewali et al. 2015  **Latino/Hispanic (2)**  Holmes & Marcelli 2014  Perera et al. 2020 | **All migrants (1)**  Azagba et al. 2019  **African (1)**  Bingham et al. 2016 | **All migrants (1)**  Meshefedjian et al. 2014 [Unadjusted]  **Asian (3)**  Bayog & Waters 2018  Jonnalagadda & Diwan 2005  Parikh et al. 2009  **Latino/Hispanic (1)**  Echeverria et al. 2013 | **Asian (1)**  Hu et al. 2010 | **All migrants (2)**  Koya & Egede 2007 [Unadjusted]  Lear et al. 2009  **Asian (3)**  Chou et al. 2010 Kuerban 2016  Talegawkar et al. 2016 |
|  | **Men** | **All migrants (2)**  Constantine et al. 2013  Li & Wen 2015  **Asian (1)**  Patel et al. 2018 | **All migrants (1)**  Lopez-Gonzalez et al. 2005  **African (1)**  Hyman et al. 2008  **Asian (2)**  Maxwell et al. 2007  Riosmena et al. 2017  **Latino/Hispanic (2)**  Maxwell et al. 2005  Riosmena et al. 2017 | **All migrants (1)**  Azagba et al. 2019  **Asian (1)**  Taylor et al. 2007 | **Asian (4)**  An et al. 2008  Hislop et al. 2008  Maxwell et al. 2005  Riosmena et al. 2017  **Latino/Hispanic (1)**  Bostean et al. 2017 | **Asian (2)**  Glenn et al. 2009  Juon et al. 2003 | **All migrants (2)**  Koya & Egede 2007  Pampel et al. 2020  **Latino/Hispanic (1)**  Riosmena et al. 2013 |
|  | **Women** | **All migrants (3)**  Constantine et al. 2013  Koya & Egede 2007  Wong & Wong 2003 [Unadjusted]  **African (1)**  Hyman et al. 2008  **Asian (3)**  An et al. 2008  Coronado et al. 2008  Patel et al. 2018 | **All migrants (3)**  Azagba et al. 2019  Li & Wen 2015  Lopez-Gonzalez et al. 2005  **Asian (3)**  Maxwell et al. 2005  Wong et al. 2011  Riosmena et al. 2017  **Latino/Hispanic (2)**  Maxwell et al. 2005  Riosmena et al. 2017 | **Latino/Hispanic (1)**  Riosmena et al. 2017 | **Asian (1)**  Maxwell et al. 2005 |  | **All migrants (2)**  Pampel et al. 20120  Wong & Wong 2003 [Adjusted]  **Latino/Hispanic (1)**  Bostean et al. 2017 |
| **Europe (Finland, France, Germany, Ireland, Netherlands, Spain, UK)** | **All** | **All migrants (1)**  Sordo et al. 2015 [Unadjusted]  **Eastern European (1)**  Kabir et al. 2008 | **African (1)**  Brathwaite et al. 2017  **Latino/Hispanic (2)**  Canfield et al. 2017  Sordo et al. 2015  **Middle Eastern (1)**  Sordo et al. 2015  **Other European (1)**  Sordo et al. 2015 |  | **Eastern European (1)**  Sordo et al. 2015 |  | **All migrants (2)**  Gazard et al. 2015  Sordo et al. 2015 [Adjusted] |
|  | **Men** | **African (1)**  Salama et al. 2018  **Middle Eastern (1)**  Reiss et al. 2014 | **African (1)**  Khlat et al. 2019 | **Eastern European (1)**  Salama et al. 2018 | **Eastern European (1)**  Reiss et al. 2015  **Middle Eastern (4)**  Reeske et al. 2009  Reiss et al. 2014  Reiss et al. 2015  Salama et al. 2018 | **Middle Eastern (1)**  Reeske et al. 2009 | **Middle Eastern (1)**  Reiss et al. 2014 |
|  | **Women** | **Eastern European (1)**  Jayaweera & Quigley 2010 [Unadjusted]  Salama et al. 2018  **Middle Eastern (2)**  Reiss et al. 2014  Reiss et al. 2015 | **All migrants (1)**  Jayaweera & Quigley 2010 [Adjusted]  **African (1)**  Khlat et al. 2019  **Eastern European (1)**  Reiss et al. 2015  **Middle Eastern (2)**  Reiss et al. 2014  Salama et al. 2018 |  | **Middle Eastern (1)**  Reiss et al. 2014 |  | **Middle Eastern (1)**  Reeske et al. 2009 |
| **Oceania (Australia)** | **All** | **All migrants (1)**  Joshi et al. 2018 |  |  |  |  | **Asian (1)**  Tran et al. 2015 |
|  | **Men** |  |  |  | **Asian (1)**  Brock et al. 2001 |  | **Asian (1)**  Jin et al. 2017 |
|  | **Women** |  |  |  |  |  | **Asian (1)**  Jin et al. 2017 |
| **Middle East**  **(Israel)** | **All** | **African (1)**  Leshem & Dessie-Navon 2018 |  |  |  |  |  |

**Table 8. Smoking studies with native-born reference**

| **Region** | **Sex** | **Convergence** | **Divergence** | **Unclear** |
| --- | --- | --- | --- | --- |
| **North America**  **(USA, Canada)** | **All** | **All migrants (1)**  Lopez-Gonzalez et al. 2005  Meshefedjian et al. 2014 [Adjusted]  Singh & Siahpush 2002  **African/Caribbean (1)**  Brown et al. 2018  **Latino/Hispanic (1)**  Perera et al. 2020 | **All migrants (1)**  Meshefedjian et al. 2014 [Unadjusted]  **Asian (1)**  Bayog & Waters 2018  **Latino/Hispanic (1)**  Echeverria et al. 2013 | **All migrants (1)**  Lear et al. 2009 |
|  | **Men** | **All migrants (1)**  Lopez-Gonzalez et al. 2005  **Asian (1)**  Riosmena et al. 2017 | **Latino/Hispanic (1)**  Bostean et al. 2017  Riosmena et al. 2017 | **All migrants (1)**  Pampel et al. 2020 |
|  | **Women** | **All migrants (1)**  Azagba et al. 2019  Lopez-Gonzalez et al. 2005  **Asian (1)**  Riosmena et al. 2017  **Latino/Hispanic (1)**  Riosmena et al. 2017 |  | **All migrants (1)**  Pampel et al. 2020  **Latino/Hispanic (1)**  Bostean et al. 2017 |
| **Europe (Finland, France, Germany, Ireland, Netherlands, Spain, UK)** | **All** | **All migrants (1)**  Sordo et al. 2015 [Unadjusted]  **Latino/Hispanic (1)**  Sordo et al. 2015  **Middle Eastern (1)**  Sordo et al. 2015 | **Eastern European (1)**  Sordo et al. 2015 | **All migrants (2)**  Gazard et al. 2015  Sordo et al. 2015 [Adjusted] |
|  | **Men** | **African (1)**  Khlat et al. 2019 |  |  |
|  | **Women** | **African (1)**  Khlat et al. 2019 |  |  |

**Alcohol use (31 studies)**

**Table 9. All alcohol use studies (regular alcohol use, unless otherwise specified)**

| **Region** | **Sex** | **Increased risk** | **Non-significant increased risk** | **No difference**  **(i.e., OR=1.00)** | **Non-significant decreased risk** | **Decreased risk** | **No trend** |
| --- | --- | --- | --- | --- | --- | --- | --- |
| **North America**  **(USA, Canada)** | **All** | **Asian (2)**  Park et al. 2014  Talegawkar et al. 2016  **Latino/Hispanic (1)**  Chartier et al. 2017  [Alcohol dependence symptoms] | **All migrants (3)**  Johnson et al. 2002 [Unadjusted]  Lopez-Gonzalez et al. 2005  Meshefedjian et al. 2014 [Unadjusted]  **Asian (1)**  Lo et al. 2014 | **All migrants (1)**  Meshefedjian et al. 2014 [Adjusted]  **African (1)**  Bingham et al. 2016  **Asian (1)**  Parikh et al. 2009 | **Latino/Hispanic (1)**  Cano et al. 2017  Borges et al. 2006 [Unadjusted] | **Asian (1)**  So & Wong 2006 | **All migrants (1)**  Johnson et al. 2002 [Adjusted]  **Asian (2)**  Agic et al. 2016 [Heavy alcohol use]  Bharmal et al. 2015 [Heavy alcohol use]  **Latino/Hispanic (1)**  Borges et al. 2006 [Adjusted]  **European (1)**  Agic et al. 2016 [Heavy alcohol use] |
|  | **Men** | **All migrants (1)**  Li & Wen 2015 [Heavy alcohol use] | **All migrants (1)**  Lopez-Gonzalez et al. 2005 [Regular/heavy alcohol use]  **Asian (1)**  Yang et al. 2007 |  | **Latino/Hispanic (1)**  Castañeda et al. 2019 | **Asian (1)**  Kim et al. 2014 [Heavy alcohol use] |  |
|  | **Women** | **All migrants (1)**  Li & Wen 2015 [Heavy alcohol use]  **Latino/Hispanic (1)**  Ertl et al. 2018 | **All migrants (1)**  Lopez-Gonzalez et al. 2005 [Regular/heavy alcohol use]  **Asian (1)**  Wong et al. 2011  **Latino/Hispanic (2)**  Castañeda et al. 2019  Hardie et al. 2013 |  | **Asian (1)**  Wong et al. 2011 |  | **Asian (1)**  Yang et al. 2007 |
| **Europe (Finland, Germany, Netherlands, Norway, Spain, UK)** | **All** |  | **All migrants (1)**  Gazard et al. 2015 [Heavy alcohol use]  **Eastern European (1)**  Sordo et al. 2015 [Heavy alcohol use]  **Latino/Hispanic (1)**  Sordo et al. 2015 [Heavy alcohol use]  **Middle Eastern (1)**  Sordo et al. 2015 [Heavy alcohol use] |  | **Latino/Hispanic (1)**  Canfield et al. 2017 [Regular/heavy alcohol use]  **Middle Eastern (1)**  Amundsen 2012  **Other European (1)**  Sordo et al. 2015 [Heavy alcohol use] |  | **Latino/Hispanic (1)**  Tortajada et al. 2010 |
|  | **Men** | **African (1)**  Addo et al. 2018 | **Middle Eastern (1)**  Salama et al. 2018 [Heavy alcohol use] |  | **Eastern European (1)**  Salama et al. 2018 [Heavy alcohol use] | **Middle Eastern (1)**  Amundsen 2012 |  |
|  | **Women** | **African (1)**  Addo et al. 2018 | **All migrants (1)**  Jayaweera & Quigley 2010 |  | **Middle Eastern (1)**  Salama et al. 2018 [Heavy alcohol use] | **Eastern European (1)**  Salama et al. 2018 [Heavy alcohol use] |  |
| **Australia** | **All** |  | **Asian (1)**  Brock et al. 2001 |  |  |  |  |

**Table 10. Alcohol use studies with native-born reference**

| **Region** | **Sex** | **Convergence** | **Divergence** | **Unclear** |
| --- | --- | --- | --- | --- |
| **North America**  **(USA, Canada)** | **All** | **All migrants (1)**  Johnson et al. 2002 [Unadjusted]  Lopez-Gonzalez et al. 2005 [Regular/heavy alcohol use]  Meshefedjian et al. 2014 [Unadjusted]  **Asian (1)**  Lo et al. 2014 | **Latino/Hispanic (1)**  Borges et al. 2006 [Unadjusted] | **All migrants (1)**  Meshefedjian et al. 2014 [Adjusted] |
|  | **Men** | **All migrants (1)**  Lopez-Gonzalez et al. 2005 [Regular/heavy alcohol use]  **Latino/Hispanic (1)**  Castañeda et al. 2019 |  |  |
|  | **Women** | **All migrants (1)**  Lopez-Gonzalez et al. 2005 [Regular/heavy alcohol use]  **Latino/Hispanic (1)**  Castañeda et al. 2019 |  |  |
| **Europe (Finland, France, Germany, Ireland, Netherlands, Spain, UK)** | **All** | **All migrants (1)**  Gazard et al. 2015  **Middle Eastern (1)**  Sordo et al. 2015 [Heavy alcohol use] | **Eastern European (1)**  Sordo et al. 2015 [Heavy alcohol use]  **Other European (1)**  Sordo et al. 2015 [Heavy alcohol use]  **Latino/Hispanic (1)**  Sordo et al. 2015 [Heavy alcohol use] | **All migrants (1)**  Sordo et al. 2015 [Heavy alcohol use] |

**Drug use (10 studies)**

**Table 11. All drug use studies (all drugs, unless otherwise specified)**

| **Region** | **Sex** | **Increased risk** | **Non-significant increased risk** | **No difference**  **(i.e., OR=1.00)** | **Non-significant decreased risk** | **Decreased risk** | **No trend** |
| --- | --- | --- | --- | --- | --- | --- | --- |
| **North America**  **(USA, Canada)** | **All** | **Latino/Hispanic (1)**  Cano 2019 [Opioids] | **All migrants (2)**  Kolar et al. 2008 [Cannabis]  Johnson et al. 2002 | **Asian (1)**  So & Wong 2006 |  | **All migrants (1)**  Yang 2019 |  |
|  | **Men** | **Latino/Hispanic (1)**  Levy et al. 2005 [Other] |  |  |  |  |  |
| **Europe (Finland, Spain, UK)** | **All** |  | **All migrants (1)**  Sordo et al. 2015  [Cannabis, other]  **Latino/Hispanic (1)**  Sordo et al. 2015  [Cannabis, other]  **Eastern European (1)**  Sordo et al. 2015 [Cannabis]  **Other European (1)**  Sordo et al. 2015 [Cannabis, other]  **Middle Eastern (1)**  Salama et al. 2018  [Cannabis, other] | **All migrants (1)**  Gazard et al. 2015  **Eastern European (1)**  Sordo et al. 2015 [Other] |  | **Latino/Hispanic (1)**  Canfield et al. 2017 |  |
|  | **Men** |  | **Eastern European (1)**  Salama et al. 2018 [Cannabis]  **Middle Eastern (1)**  Salama et al. 2018  [Cannabis] |  |  |  |  |
|  | **Women** | **Eastern European (1)**  Salama et al. 2018 [Cannabis] |  |  |  |  |  |

**Table 12. Drug use studies with native-born reference**

| **Region** | **Sex** | **Convergence** | **Divergence** | **Unclear** |
| --- | --- | --- | --- | --- |
| **North America**  **(USA, Canada)** | **All** | **All migrants (2)**  Kolar et al. 2008 [Cannabis]  Johnson et al. 2002 |  |  |
| **Europe (Finland, Spain, UK)** | **All** | **All migrants (1)**  Sordo et al. 2015 [Cannabis, other]  **Latino/Hispanic (1)**  Sordo et al. 2015 [Cannabis, other]  **Eastern European (1)**  Sordo et al. 2015 [Cannabis]  **Other European (1)**  Sordo et al. 2015 [Cannabis, other] |  | **All migrants (1)**  Gazard et al. 2015  **Eastern European (1)**  Sordo et al. 2015 [Other] |

**Substance use dependence/diagnosis (10 studies)**

**Table 13. All substance use studies (all substances, unless otherwise specified)**

| **Region** | **Sex** | **Increased risk** | **Non-significant increased risk** | **No difference**  **(i.e., OR=1.00)** | **Non-significant decreased risk** | **Decreased risk** | **No trend** |
| --- | --- | --- | --- | --- | --- | --- | --- |
| **North America (USA, Canada)** | **All** | **All migrants (1)**  Patterson et al. 2013  **Black/Caribbean (1)**  Lacey et al. 2015 [Drug use disorder] | **All migrants (2)**  Breslau et al. 2007  Salas-Wright et al. 2018  **Asian (1)**  Breslau et al. 2006  **Black/Caribbean (2)**  Lacey et al. 2015 [Substance, alcohol use disorder]  Lacey et al. 2016  **Latino/Hispanic (1)**  Borges et al. 2006 [Adjusted, alcohol use disorder] | **Latino/Hispanic (1)**  Blanco et al. 2013 [Drug use disorder] |  |  | **Latino/Hispanic (1)**  Borges et al. 2006 [Unadjusted, alcohol use disorder] |
|  | **Men** |  | **Latino/Hispanic (1)**  Alegria et al. 2007 |  |  |  | **Asian (1)**  Takeuchi et al. 2007 |
|  | **Women** |  | **Asian (1)**  Takeuchi et al. 2007  **Latino/Hispanic (1)**  Alegria et al. 2007 |  |  |  |  |

**Table 14. Substance use studies with native-born reference**

| **Region** | **Sex** | **Convergence** | **Divergence** | **Unclear** |
| --- | --- | --- | --- | --- |
| **North America**  **(USA, Canada)** | **All** | **All migrants (2)**  Breslau et al. 2007  Salas-Wright et al. 2018  **Asian (1)**  Breslau et al. 2006  **Black/Caribbean (1)**  Lacey et al. 2016  **Latino/Hispanic (1)**  Borges et al. 2006 [Adjusted, alcohol use disorder] |  | **Latino/Hispanic (1)**  Blanco et al. 2013 [Drug use disorder]  Borges et al. 2006 [Unadjusted, alcohol use disorder] |
|  | **Men** | **Latino/Hispanic (1)**  Alegria et al. 2007 |  | **Asian (1)**  Takeuchi et al. 2007 |
|  | **Women** | **Asian (1)**  Takeuchi et al. 2007  **Latino/Hispanic (1)**  Alegria et al. 2007 |  |  |

**Physical activity (49 studies)**

**Table 15. All/unspecified physical inactivity studies**

| **Region** | **Sex** | **Increased risk** | **Non-significant increased risk** | **No difference**  **(i.e., OR=1.00)** | **Non-significant decreased risk** | **Decreased risk** | **No trend** |
| --- | --- | --- | --- | --- | --- | --- | --- |
| **North America**  **(USA, Canada)** | **All** | **All migrants (1)**  Terasaki et al. 2017  **Asian (3)**  Li & Hummer 2015  Talegawkar et al. 2016  Taylor et al. 2007  **Latino/Hispanic (1)**  Perera et al. 2020 | **All migrants (1)**  Vargas-Bustamante 2013  **African (1)**  Sewali et al. 2015  **Asian (1)**  Osypuk et al. 2009  **Latino/Hispanic (2)**  Murillo et al. 2015  Osypuk et al. 2009 | **African (1)**  Bingham et al. 2016  **Asian (1)**  Hislop et al. 2008 | **All migrants (1)**  Dogra et al. 2010  **Asian (1)**  Bayog & Waters 2018 | **Asian (2)**  Jonnalagadda & Diwan 2005  Parikh et al. 2009  **Latino/Hispanic (1)**  Creighton et al. 2012 | **All migrants (1)**  Terasaki et al. 2017  **Asian (1)**  Bharmal et al. 2015 |
|  | **Men** |  | **Latino/Hispanic (1)**  Kershaw et al. 2016 |  | **All migrants (1)**  Dogra et al. 2010  **African (1)**  Commodore-Mensah et al. 2018  **Non-English-speaking-origin (1)**  Zan & Fan 2018 |  |  |
|  | **Women** |  |  | **Latino/Hispanic (1)**  Kershaw et al. 2016  **Non-English-speaking-origin (1)**  Zan & Fan 2018 | **All migrants (1)**  Dogra et al. 2010  **African (1)**  Commodore-Mensah et al. 2018  **Asian (1)**  Coronado et al. 2008  **Latino/Hispanic (1)**  Evenson et al. 2004 [Adjusted] | **Latino/Hispanic (1)**  Evenson et al. 2004 [Unadjusted] |  |
| **Europe (France, Germany, UK)** | **All** |  |  | **Middle Eastern (1)**  Koca & Lapa 2014 | **Middle Eastern (1)**  Koca & Lapa 2014 |  |  |
|  | **Men** |  |  | **African (1)**  Mejean et al. 2009 |  |  |  |
|  | **Women** |  |  |  |  |  |  |
| **Oceania (Australia)** | **All** |  | **Non-English-speaking-origin (1)**  Joshi et al. 2017 | **Asian (1)**  Tran et al. 2015 |  | **Asian (1)**  Brock et al. 2001 | **English-speaking-origin (1)**  Joshi et al. 2017 |
|  | **Men** |  | **Asian (1)**  Jin et al. 2018 |  |  |  |  |
|  | **Women** |  |  |  | **Asian (1)**  Jin et al. 2018 |  |  |
| **Middle East**  **(Israel)** | **All** |  | **African (1)**  Leshem & Dessie-Navon 2018 |  |  |  |  |

**Table 16. Leisure-time physical inactivity (i.e., exercise) studies**

| **Region** | **Sex** | **Increased risk** | **Non-significant increased risk** | **No difference**  **(i.e., OR=1.00)** | **Non-significant decreased risk** | **Decreased risk** | **No trend** |
| --- | --- | --- | --- | --- | --- | --- | --- |
| **North America**  **(USA, Canada)** | **All** | **All migrants (1)**  Mahmood et al. 2019 | **All migrants (1)**  Meshefedjian et al. 2014  **African (1)**  Tremblay et al. 2006  **Asian (1)** Lear et al. 2009  **European (1)**  Lear et al. 2009 | **Asian (1)**  Tremblay et al. 2006  **European (1)**  Tremblay et al. 2006  **Latino/Hispanic (1)**  Tremblay et al. 2006 | **All migrants (3)**  Basu & Insler 2019  Dogra et al. 2010  Tremblay et al. 2006  **African/Caribbean (1)**  Brown et al. 2018  **Asian (1)**  Tremblay et al. 2006  **Latino/Hispanic (2)**  Creighton et al. 2012  Murillo et al. 2015  **Middle-Eastern (1)**  Tremblay et al. 2006 | **All migrants (4)**  Basu & Insler 2019  Gaskins et al. 2012  Koya & Egede 2007  Li & Wen 2013  **Asian (1)**  Afable et al. 2016  **Latino/Hispanic (1)**  Echeverria et al. 2013 | **Latino/Hispanic (1)**  Crespo et al. 2001 |
|  | **Men** |  | **African (1)**  Tremblay et al. 2006 | **Asian (1)**  Tremblay et al. 2006  **European (1)**  Tremblay et al. 2006  **Latino/Hispanic (1)**  Tremblay et al. 2006 | **All migrants (2)**  Dogra et al. 2010  Tremblay et al. 2006  **Asian (1)**  Tremblay et al. 2006  **Non-English-speaking-origin (1)**  Zan & Fan 2018 | **All migrants (1)**  Koya & Egede 2007  **Asian (1)**  Kandula & Lauderdale 2005 | **Latino/Hispanic (1)**  Crespo et al. 2001 |
|  | **Women** |  | **Middle-Eastern (1)**  Tremblay et al. 2006 | **African (1)**  Tremblay et al. 2006  **Asian (1)**  Tremblay et al. 2006  **European (1)**  Tremblay et al. 2006  **Latino/Hispanic (1)**  Tremblay et al. 2006 | **All migrants (4)**  Dogra et al. 2010  Koya & Egede 2007  Tremblay et al. 2006  Yang 2019  **Non-English-speaking-origin (1)**  Zan & Fan 2018  **Latino/Hispanic (1)**  Crespo et al. 2001 [Unadjusted] | **All migrants (1)**  Wong & Wong 2003  **Asian (2)**  Choi et al. 2008  Kandula & Lauderdale 2005 | **Latino/Hispanic (1)**  Crespo et al. 2001 [Adjusted] |
| **Europe (France, Netherlands, Sweden, UK)** | **All** |  | **African (1)**  de Munter et al. 2013 | **African (1)**  de Munter et al. 2013  **Asian (1)**  de Munter et al. 2013 | **African (1)**  de Munter et al. 2013  **Asian (1)**  de Munter et al. 2013 |  | **African (1)**  de Munter et al. 2013  **Asian (1)**  de Munter et al. 2013 |
|  | **Men** |  |  |  | **All migrants (1)**  Dawson et al. 2005 |  | **African (1)**  Mejean et al. 2009 |
|  | **Women** |  |  |  |  | **All migrants (1)**  Dawson et al. 2005 |  |
| **Oceania (Australia)** | **Women** |  |  | **Asian (1)**  Lee et al. 2007 |  |  |  |

**Table 17. Non-leisure-time physical inactivity (i.e., work, travel, household tasks) studies**

| **Region** | **Sex** | **Increased risk** | **Non-significant increased risk** | **No difference**  **(i.e., OR=1.00)** | **Non-significant decreased risk** | **Decreased risk** | **No trend** |
| --- | --- | --- | --- | --- | --- | --- | --- |
| **North America**  **(USA, Canada)** | **All** | **All migrants (1)**  Basu & Insler 2019 [Work]  **Asian (1)**  Afable et al. 2016 [Work] | **All migrants (2)**  Basu & Insler 2019 [Work]  Dogra et al. 2010 [Work/travel]  **Latino/Hispanic (2)**  Ham et al. 2007 [Travel]  Murillo et al. 2015 [Work/travel} | **All migrants (1)**  Basu & Insler 2019 [Work]  **Latino/Hispanic (1)**  Ham et al. 2007 |  | **Latino/Hispanic (1)**  Creighton et al. 2013 [Work] | **Asian (1)**  Afable et al. 2016 [Travel] |
|  | **Men** |  | **All migrants (1)**  Dogra et al. 2010 [Work/travel] | **Non-English-speaking-origin (1)**  Zan & Fan 2018 [Household] | **Non-English-speaking-origin (1)**  Zan & Fan 2018 [Travel] |  |  |
|  | **Women** |  | **Asian (1)**  Kandula & Lauerdale 2005  **Latino/Hispanic (1)**  Evenson et al. 2004 [Work]  **Non-English-speaking-origin (1)**  Zan & Fan 2018 [Household] | **All migrants (1)**  Dogra et al. 2010 [Work/travel]  **Non-English-speaking-origin (1)**  Zan & Fan 2018 [Travel] | **Latino/Hispanic (1)**  Evenson et al. 2004 [Work] |  | **Asian (1)**  Kandula & Lauerdale 2005 |
| **Europe (France)** | **Men** |  |  |  |  |  | **African (1)**  Mejean et al. 2009 |

**Table 18. Physical inactivity studies with native-born reference**

| **Region** | **Sex** | **Convergence** | **Divergence** | **Unclear** |
| --- | --- | --- | --- | --- |
| **North America**  **(USA, Canada)** | **All** | **All migrants (3)**  Dogra et al. 2010 [All, general leisure-time]  Meshefedjian et al. 2014  Tremblay et al. 2006  **Asian (2)**  Bayog & Waters 2018  Osypuk et al. 2009  **Latino/Hispanic (4)**  Echeverria et al. 2013  Murillo et al. 2015  Osypuk et al. 2009  Perera et al. 2020 | **All migrants (2)**  Dogra et al. 2010 [Exercise]  Vargas-Bustamante 2013  **African (1)**  Tremblay et al. 2006  **Asian (1)**  Lear et al. 2009  **European (1)**  Lear et al. 2009 | **Latino/Hispanic (1)**  Crespo et al. 2001 |
|  | **Men** | **All migrants (2)**  Dogra et al. 2010 [All, general leisure-time]  Tremblay et al. 2006  **Asian (1)**  Kandula & Lauderdale 2005  **Non-English-speaking-origin (1)**  Zan & Fan 2018 | **All migrants (1)**  Dogra et al. 2010 [Exercise]  **African (1)**  Tremblay et al. 2006  **Latino/Hispanic (1)**  Kershaw et al. 2016 |  |
|  | **Women** | **All migrants (2)**  Dogra et al. 2010 [All, general leisure-time]  Tremblay et al. 2006  **Asian (1)**  Kandula & Lauderdale 2005  **Non-English-speaking-origin (1)**  Zan & Fan 2018 | **All migrants (1)**  Dogra et al. 2010 [Exercise] | **African (1)**  Tremblay et al. 2006  **Latino/Hispanic (1)**  Kershaw et al. 2016 |
| **Europe (Netherlands, UK)** | **All** | **African (1)**  de Munter et al. 2013  **Asian (1)**  de Munter et al. 2013 | **African (1)**  de Munter et al. 2013 | **African (1)**  de Munter et al. 2013  **Asian (1)**  de Munter et al. 2013 |
| **Other** | **All** |  | **African (1)**  Leshem & Dessie-Navon 2018  **Non-English-speaking-origin (1)**  Joshi et al. 2017 | **English-speaking-origin (1)**  Joshi et al. 2017 |

**Diet (17 studies)**

**Table 19. All diet studies with daily energy intake**

| **Region** | **Sex** | **Increased risk** | **Non-significant increased risk** | **No difference**  **(i.e., OR=1.00)** | **Non-significant decreased risk** | **Decreased risk** | **No trend** |
| --- | --- | --- | --- | --- | --- | --- | --- |
| **North America (USA, Canada)** | **All** |  | **All migrants (1)**  Basu & Insler 2019  **African/Caribbean (1)**  Brown et al. 2018  **Asian (1)**  Lear et al. 2009  **European (1)**  Lear et al. 2009 |  |  | **Asian (1)**  Talegawkar et al. 2016 |  |
|  | **Women** |  |  | **Asian (1)**  Tseng & Fang 2011/  Tseng et al. 2015 |  |  |  |
| **Europe (Spain, UK)** | **All** | **African/Caribbean (1)**  Goff et al. 2015  **Latino/Hispanic (1)**  Marin-Guerrero et al. 2016 |  |  |  |  |  |
| **Oceania (Australia)** | **Women** |  | **Asian (1)**  Lee et al. 2007 |  |  |  |  |
| **Middle East (Israel)** | **All** | **African (1)**  Leshem & Dessie-Navon 2018 |  |  |  |  |  |

**Table 20. All diet studies with healthy diet scores**

| **Region** | **Sex** | **Increased risk** | **Non-significant increased risk** | **No difference**  **(i.e., OR=1.00)** | **Non-significant decreased risk** | **Decreased risk** | **No trend** |
| --- | --- | --- | --- | --- | --- | --- | --- |
| **North America (USA, Canada)** | **All** | **African/Caribbean (2)**  Brown et al. 2018  Huffman et al. 2014  **Latino/Hispanic (2)**  Perera et al. 2020  Van Hook et al. 2018 | **African/Caribbean (2)**  Brown et al. 2018  Huffman et al. 2014  **Latino/Hispanic (1)**  Marin-Guerrero et al. 2015 | **Latino/Hispanic (1)**  Sofianou et al. 2011 |  |  |  |
|  | **Men** |  | **Latino/Hispanic (1)**  Kershaw et al. 2016 |  |  |  |  |
|  | **Women** |  | **Latino/Hispanic (1)**  Kershaw et al. 2016 |  |  |  |  |
| **Europe (Netherlands, Spain)** | **All** | **African (1)**  Delisle et al. 2009 | **Latino/Hispanic (1)**  Marin-Guerrero et al. 2016 |  |  |  |  |
|  | **Men** |  |  | **African/Caribbean (1)**  Nicolaou et al. 2006  **Asian (1)**  Nicolaou et al. 2006 |  |  |  |
|  | **Women** |  |  | **African/Caribbean (1)**  Nicolaou et al. 2006  **Asian (1)**  Nicolaou et al. 2006 |  |  |  |

**Table 21. Diet studies with native-born reference**

| **Region** | **Sex** | **Convergence** | **Divergence** | **Unclear** |
| --- | --- | --- | --- | --- |
| **North America**  **(USA, Canada)** | **All** | **Asian (1)**  Lear et al. 2009  **European (1)**  Lear et al. 2009 | **Latino/Hispanic (1)**  Perera et al. 2020 |  |
|  | **Men** |  | **Latino/Hispanic (1)**  Kershaw et al. 2016 |  |
|  | **Women** |  | **Latino/Hispanic (1)**  Kershaw et al. 2016 |  |
